# Ectopic CD11c Drives SMAD3-Mediated Aberrant Antigen Presentation and Epithelial–Mesenchymal Transition in Esophageal Squamous Cell Carcinoma

**DOI:** 10.34133/cancomm.0014

**Published:** 2026-03-06

**Authors:** Han Liao, Xuan Zhao, Liping Chen, Qingyi Liu, Chenying Li, Kai Li, Yiyi Xi, Yanrong Shen, Wen Tan, Chen Wu, Dongxin Lin

**Affiliations:** ^1^ Department of Etiology and Carcinogenesis, National Cancer Center/National Clinical Research Center/Cancer Hospital, Chinese Academy of Medical Sciences (CAMS) and Peking Union Medical College (PUMC), Beijing, P. R. China.; ^2^ Key Laboratory of Cancer Genomic Biology, Chinese Academy of Medical Sciences and Peking Union Medical College, Beijing, P. R. China.; ^3^ Collaborative Innovation Center for Cancer Personalized Medicine, Nanjing Medical University, Nanjing, Jiangsu, P. R. China.; ^4^ Chinese Academy of Medical Sciences Oxford Institute, Chinese Academy of Medical Sciences, Beijing, P. R. China.; ^5^ State Key Laboratory of Oncology in South China, Sun Yat-sen University Cancer Center, Guangzhou, Guangdong, P. R. China.

## Abstract

**Background:** Esophageal squamous cell carcinoma (ESCC) is a highly aggressive cancer with a poor prognosis, where immune evasion plays a central role in tumor progression and resistance to therapy. The underlying mechanisms of tumor–stroma interactions remain poorly understood, despite the relationship between epithelial–mesenchymal transition (EMT) and altered immune response having been suggested. This study aimed to investigate how phenotypic shifts in ESCC tumor cells contribute to immune modulation. **Methods:** We used multiplex immunofluorescence on 4-nitroquinoline 1-oxide (4-NQO)-induced multistage mouse ESCC models to characterize the local tumor microenvironment. Additionally, we integrated multiomics datasets, including spatial transcriptomics, single-cell RNA sequencing, and proteomics, from multistage human esophageal samples to investigate the underlying molecular mechanisms. These findings were further validated through in vitro cell line experiments and in vivo therapeutic models. **Results:** We identified an ESCC cell cluster with ectopic expression of CD11c (also known as integrin alpha X), in both mice and humans, probably formed via tumor protein p53 (*TP53*) inactivation, causing cancer cells to escape immune killing and gain malignant phenotypes. CD11c impaired cancer cell antigen presentation and fostered EMT through up-regulation of mothers against decapentaplegic homolog 3 (SMAD3) phosphorylation in human ESCC cell lines. Mechanistically, CD11c activated SMAD3 to suppress costimulatory factors CD80/CD86 and augmented immunosuppressive CD4^+^ T cell responses through aberrant major histocompatibility complex class II-mediated antigen presentation. Evaluation in humanized mouse models further confirmed that CD11c overexpression in ESCC resulted in immune evasion, tumor metastasis, and resistance to anti-programmed death ligand 1 (PD-L1) therapy, but could be rescued by combined treatment with anti-phospho-SMAD3. **Conclusions:** This study reveals a mechanism by which ectopic CD11c expression causes immunosuppression and contributes to the acquisition of malignant phenotypes in ESCC. Targeting the CD11c–SMAD3 axis may enhance the efficacy of existing immunotherapies, potentially improving the treatment outcomes of ESCC patients.

## Background

In the tumor microenvironment (TME), effective antigen presentation (AP) and normal immune cell function are essential for the immune system to efficiently recognize and eradicate cancer cells [1,2]. However, cancer cells develop various strategies to evade immune surveillance by harnessing adaptive immunity, enabling them to resist therapies aimed at restoring effective immune responses [3–5]. In some cases, the local TME becomes dysfunctional before tumor formation, providing niches for cancer development [5–7]. Carcinoma cells acquire epithelial–mesenchymal transition (EMT), a highly aggressive phenotype that stimulates local stromal cells to assist in tumor invasion and migration [8]. Beyond its role in invasion and metastasis, EMT is increasingly recognized as associated with altered immune states, including reduced immune surveillance and therapeutic responsiveness [9–11]. Evidence suggests that EMT-associated transcriptional programs do not merely accompany immune dysfunction but can actively contribute to immune evasion and resistance to immunotherapy [12,13].

Consistent with this concept, our previous studies on multistage human esophageal squamous cell carcinoma (ESCC) revealed that precancerous and cancerous epithelial cells continuously interact with each other and other cell types in the local TME, reshaping disease developmental patterns [14,15]. Moreover, our previous studies have identified 8 epithelial subpopulations in ESCC and precancerous cells, among which mesenchymal-like and antigen-presenting-related epithelial cells exhibited concurrent gene expression patterns, suggesting an important tumor-intrinsic phenomenon [14,16]. Although the association has been described at the tissue or cellular population level, whether EMT and AP dysfunction coexist independently or are integrated intrinsically in epithelial cells remains unclear. If the latter is true, revealing the key underlying molecules would be crucial for understanding the development of ESCC and other epithelial carcinomas, potentially facilitating the discovery of therapeutic targets.

Human ESCC development, like many other carcinomas, follows a stepwise paradigm from normal (NOR), inflammation (INF), low-grade intraepithelial neoplasia (LGIN), high-grade intraepithelial neoplasia (HGIN), to invasive carcinoma [17], and these processes can be mimicked in mice treated with chemical carcinogens such as 4-nitroquinoline 1-oxide (4-NQO) [18]. Increasing grades of dysplasia are strongly associated with an increased risk of ESCC mortality, and the 5-year survival rate remains at approximately 30% [19,20] owing to metastasis and resistance to chemoradiotherapy and immunotherapy [21,22]. Although immune checkpoint blockade (ICB) has shown promise in several solid tumors, its efficacy in ESCC remains limited, underscoring the need to understand tumor-intrinsic mechanisms that confer immune escape and therapeutic resistance, as they may be promising in other cancer types but not in ESCC [23]. Moreover, the mechanism whereby cancer cells avoid immune killing remains unknown. Understanding the EMT crosstalk within TME in cancer is now proving useful in the development of prognostic markers, therapeutic indications, and novel therapy combinations. Therefore, uncovering the relationship between ESCC progression and immunotherapy resistance is necessary to develop diagnostic and therapeutic strategies.

In the present study, we investigated how epithelial cell plasticity intersects with immune regulation during ESCC development. Using collected multistage esophageal lesions and adjacent normal tissue samples from mice, we spatiotemporally delineated local ESCC TMEs and attempted to investigate tumor–stroma interactions. We identified a specific epithelial cell subcluster that enhances ESCC malignancy, as indicated by the concurrent manifestation of abnormal AP activity and an EMT phenotype in CD11c^+^ epithelial cells. Our results demonstrated that during malignant transition, abnormal CD11c^+^ esophageal epithelial cells emerged, expanded, and exerted aberrant AP and EMT phenotypes, thereby contributing to immune evasion and reduced sensitivity to programmed death ligand 1 (PD-L1) blockade. This work provides a mechanistic framework for understanding EMT-associated immune resistance in ESCC and highlights potential vulnerabilities for improving immunotherapeutic responses.

## Methods

### Mouse ESCC induction and sample collection

Animal experiments in this study were conducted in conformity with the approved ethical protocols and guidelines of the Institutional Animal Care and Use Committee of the Chinese Academy of Medical Sciences (NCC2021A271). We established 4-NQO-induced mouse ESCC models as previously described [18]. Briefly, 180 female C57BL/6 mice (7 weeks old; Research Resource Identifier [RRID]: IMSR_JAX:000664) were administered with 4-NQO (Sigma-Aldrich, Cat. #1N8141) in drinking water (100 μg/ml) for 16 weeks. The esophagus of each mouse was collected and either formalin-fixed and paraffin-embedded or frozen at −80 °C.

### Clinical samples

For multiplex immunofluorescence (mIF) staining, surgically resected human ESCC and adjacent normal tissues were collected from patients (*n* = 11) at the Cancer Hospital, Chinese Academy of Medical Sciences, with approval from the Institutional Review Boards of the Cancer Hospital, Chinese Academy of Medical Sciences (NCC2022C141). All specimens were formalin-fixed and paraffin-embedded. Human peripheral blood mononuclear cells (PBMCs) (Schbio Biotech, Cat. #PBMNC100C) for humanized mouse model establishment were commercially acquired.

### mIF assay and data processing

Mouse esophageal tissues with NOR, INF, LGIN, HGIN, and ESCC lesions were used to construct a tissue microarray (TMA). We performed mIF staining on adjacent TMA serial sections. Two panels of antibodies were used: Panel 1 included antibodies against CD68, CD206, CD11c, or pan-cytokeratin (panCK), and Panel 2 (tumor-infiltrating lymphocyte panel) included antibodies against CD3, CD4, CD8, or forkhead box P3 (FOXP3). We performed the staining procedure according to a previously published study based on the modified Opal Multiplexed IHC assay [24]. Nuclei were stained with 4′,6-diamidino-2-phenylindole (DAPI), and the details of the mIF antibodies are shown in Table S1. We acquired tissue H&E staining images of adjacent slices simultaneously, and each image was reviewed by 2 pathologists for histopathological examination. Multispectral images were scanned using the Vectra Polaris Automated Quantitative Pathology Imaging System and then unmixed to remove autofluorescence by inForm software (AKOYA, version 2.6, RRID: SCR_019155; https://www.akoyabio.com/phenoimager/inform-tissue-finder/) as described. After quality control, we generated a dataset of 123 multispectral images (52 NOR, 27 INF, 21 LGIN, 10 HGIN, and 13 ESCC) for further analysis. Based on tissue histopathology and manual inspection, inForm software was used to define the epithelium and stroma compartments within each tissue. The inForm software was then trained on cell segmentation based on DAPI nuclear staining. The intensity of each marker expression was used to calculate the thresholds for marker positivity, thus phenotyping all cells accordingly.

We calculated the marker intensity thresholds using Otsu’s method, which is used for robust discrimination between true positive signals and background fluorescence. Spatial distribution distances were estimated for each type of cell using the R package “phenoptr” (https://akoyabio.github.io/phenoptr/). For each slice, we calculated the distance between other cell types and the nearest neighbor of the target cell type using the “find_nearest_distance” function of “phenoptr” and counted cells within a specific radius using the “count_within” function of “phenoptr”.

### Flow cytometry

Flow cytometry was performed using a BD FACSMelody instrument. The antibodies used for flow cytometry analysis are shown in Table S1. Briefly, fresh esophageal epithelial tissues from 4-NQO-treated mice or lung metastases from humanized mice were dissociated for 60 min at 37 °C by digestion with collagenase I (0.5 mg/ml) and collagenase IV (0.5 mg/ml) from Gibco, and DNase I (10 μg/ml) from Roche in RPMI-1640 medium. Dispersed cells were passed through a 70-μm strainer for single-cell suspensions and then treated with RBC lysis solution (BD Biosciences), and flow cytometry staining was performed immediately after the single-cell suspension was obtained. Cells from esophageal epithelium were stained with fluorochrome-conjugated antibodies against CD45, panCK, and CD11c, and lung metastases were stained with CD45, CD3, CD8, CD25, FOXP3, or interferon gamma (IFN-γ). We used 7-aminoactinomycin D for dead cell exclusion. Fluorescence minus one control was used to define positivity thresholds for each marker. At least 10,000 live epithelial cells were collected from each sample. Compensation was performed using single stains. To rigorously confirm the epithelial origin of CD11c expression, epithelial cells were gated as CD45^-^panCK^+^, and CD11c expression was evaluated within this subset.

For analysis of direct AP by ESCC cell lines, human CD4^+^ T cells were seeded in a 24-well plate at a density of 1.0 × 10^6^ cells per well and then cocultured with ESCC cells for 24 h. Intracellular cytokine staining for interleukin-10 (IL-10) and IFN-γ was performed following phorbol myristate acetate (PMA)/ionomycin stimulation (BD Biosciences, RRID: AB_2868893) for 4 h. After incubation, CD4^+^ T cells were collected and stained with antibodies (5 μg per immunoprecipitation) against CD4, CD25, FOXP3, IL-10, or IFN-γ. Each antibody was used at a concentration of 5 μl per 100 μl of staining volume per sample. Flow cytometry profiles were analyzed using FlowJo software (version 10.1, BD Biosciences, RRID: SCR_008520).

### Whole-genome bisulfite sequencing data analysis

We performed DNA methylation analysis based on whole-genome bisulfite sequencing of 40 esophageal samples from 14 ESCC patients, as reported previously [14]. First, we used the R package biomaRt (version 2.56.1, https://github.com/grimbough/biomaRt) to obtain information on the chromosome and transcription start site (TSS) locations of the integrin alpha X (*ITGAX*) gene. The key promoter region was considered within 500 base pairs upstream of the TSS. Next, the R package DSS (https://bioconductor.org/packages/release/bioc/html/DSS.html) was used to calculate the differential methylation sites. Finally, the methylation level of the *ITGAX* promoter in a certain sample was calculated by averaging the methylation levels of all CpG sites in the region.

### Whole-genome sequencing data analysis

We focused on the copy number change of the *ITGAX* gene in our previously published human ESCC multistage genomic data [25]. To estimate changes among histopathological stages, we calculated the absolute copy number for the *ITGAX* locus of 1,275 micro-biopsies from 42 samples with multistage lesions of ESCC. Estimated copy numbers from GISTIC (version 2.0.23, https://github.com/broadinstitute/gistic2) were used to compare the changes under different *TP53* statuses (biallelic loss, multiple mutations, one mutation, and wild type) at the clone level.

### Patient survival analysis

We performed a patient survival analysis based on bulk RNA sequencing data from 4 ESCC cohorts (total *n* = 356) using the Kaplan–Meier method and log-rank test. Cohorts 1 (*n* = 43) and 3 (*n* = 139) were from Zhang et al. [16], Cohort 2 (*n* = 94) was from Chang et al. [25], and Cohort 4 (*n* = 80) was from The Cancer Genome Atlas (https://www.cancer.gov/ccg/research/genome-sequencing/tcga, project ID: TCGA-ESCA). We first estimated the expression levels (log TPM [transcripts per million]) of specific genes in tumors using the R package ESTIMATE (version 1.0.13, https://bioinformatics.mdanderson.org/estimate/) and then applied the “surv_cutpoint” function in the R package survminer (version 0.4.9, https://github.com/kassambara/survminer) to obtain the suitable cutoffs, thus acquiring the most significant relationship with the survival probability for each factor. After the high/low groups were identified, we entered these variables into Cox proportional hazards models, where the hazard ratios (HRs) and their *P* values were calculated univariately.

### Gene set variation analysis

We used the GSVA package (version 1.42.0, https://github.com/rcastelo/GSVA) with the default parameters to estimate the activities of Hallmark pathways exported from the MSigDB R package (version 7.5.1, https://cran.r-project.org/web/packages/msigdbr/index.html). Differential pathway activity of each cluster was calculated using the limma R package (version 3.50.3, https://bioinf.wehi.edu.au/limma/). The significant differential pathways (*P* < 0.05) with high pathway activity scores were visualized using bar plots.

### Differential gene expression and gene enrichment analysis

We performed differential gene expression and subsequent gene enrichment analyses based on our previously published human ESCC multistage spatial transcriptomic data [14]. According to the *ITGAX* expression, each spot at different histological stages was classified as *ITGAX* negative or positive. Next, the differentially expressed genes between these 2 groups in the HGIN or ESCC stage of each patient were calculated, and genes significantly up-regulated in the *ITGAX*-positive group with a fold change > 2 and false discovery rate (FDR) < 0.05 were manually selected for subsequent gene enrichment analysis. Hallmark pathways from the MSigDB database were selected as the reference gene set, and the R package clusterProfiler (version 4.2.2, https://github.com/YuLab-SMU/clusterProfiler/) was applied to calculate hypermetric *P* values and visualize the results.

### Cell lines and cell culture

Human ESCC cell lines KYSE30 (RRID: CVCL_1351), KYSE150 (RRID: CVCL_1348), and KYSE510 (RRID: CVCL_1354) were generous gifts from Dr. Yutaka Shimada of Hyogo College of Medicine, Japan. The immortalized normal esophageal epithelial cell line HET-1A (RRID: CVCL_3702) and the human embryonic kidney 293 cell line HEK-293T (RRID: CVCL_0063) were purchased from the American Type Culture Collection. All cell lines were validated by DNA fingerprinting analysis and tested for the absence of mycoplasma infection. KYSE30, KYSE150, and KYSE510 cells were cultured in RPMI 1640 medium with 10% fetal bovine serum (FBS), while HET-1A and HEK-293T cells were cultured in Dulbecco’s Modified Eagle’s Medium (DMEM) with 10% FBS.

Mononuclear cells were separated from 10 ml of fresh peripheral bone marrow specimens by density gradient centrifugation using a Ficoll cushion. Naive CD4^+^ T cells were immunomagnetically isolated using CD4 microbeads (Miltenyi Biotec, Cat. #AB_2889919) and cultured in RPMI 1640 medium containing 10% FBS with Human CD3/CD28 T Cell Activator (Stemcell, Cat. #10971) and IL-2 (10 ng/ml, Proteintech). For the induction of IL-10 production, we cultured CD4^+^ T cells with IL-2 and recombinant human transforming growth factor beta 1 (rhTGFβ1; Proteintech, Cat. #HZ-1011; 5 ng/ml) for 72 h. All cell lines were incubated under 5% CO_2_ conditions. For the treatment with rhTGFβ1 (Proteintech, Cat. #HZ-1011) or transforming growth factor beta receptor I (TGFBR1) inhibitor SB505124 (Selleck, Cat. #S2186), the reagents were dissolved in phosphate-buffered saline (PBS) and added to cell culture medium at a final concentration of 10 ng/ml rhTGFβ1 or 10 μmol/l SB505124.

### Plasmids, siRNA, and cell transfection

Transient overexpression of CD11c truncated mutants or full-length CD11c was constructed by inserting the coding sequences (CDS) region into the pCMV vector, respectively; full-length SMAD3 plasmids were constructed in the pcDNA3.1 vector. All plasmids were synthesized and purchased from Tsingke Biotechnology. The transfection of plasmids was performed using Lipofectamine 3000 (Invitrogen, Cat. #L3000015) according to the manufacturer’s instructions. Briefly, 7.0 × 10^5^ cells/well were seeded in 6-well plates overnight, followed by transfection with a mixture containing plasmid and Lipofectamine 3000. Fresh medium was replaced 6 h after transfection, and cells were collected at the specified time points.

Small interfering RNAs (siRNAs) targeting human *SMAD3* were purchased from JTSBIO, and the sequences are shown in Table S2. The transfection of siRNAs was performed using jetPRIME (Polyplus, Cat. #101000046) according to the instructions of the manufacturer.

### Establishment of cell lines with altered *ITGAX* expression

Lentiviruses for stable *ITGAX*-overexpression (*ITGAX*-OE; Ubi-MCS-3FLAG-CBh-gcGFP-IRES-puromycin) or knockout (U6-sgRNA-EF1a-Cas9-FLAG-CMV-EGFP-P2A-puro) were from GeneChem as viral particles. ESCC cells (KYSE30 and KYSE150) were infected with the viruses and cultured in complete medium for 24 h, followed by selection with puromycin (Selleck, Cat. #S7417) for another 7 days. The efficiency of overexpression or knockout was examined by immunoblot analysis. The stable knockout target sequences are shown in Table S2.

### Enzyme-linked immunospot assay

CD4^+^ T cells were cultured overnight in conditional medium containing 10% FBS and IL-2 in a 24-chambered plate and then cocultured with or without ESCC cells for 24 h. CD4^+^ T cells were then collected immediately for enzyme-linked immunospot (ELISpot) assay, with the Human IL-10 ELISpot Set (BD Biosciences, Cat. #551018), to enumerate the number of IL-10-producing T cells. Briefly, CD4^+^ T cells were cultured with the functionally validated recombinant protein for 24 h in a 96-well plate coated with anti-human IL-10 at 37 °C (4.0 × 10^5^ cells in each well with one peptide pool, 2 μg of each peptide per well), and the number of T cells that were activated by the recombinant protein or coculture with ESCC cells and secreted IL-10 was detected using an ELISpot assay. In parallel, the assays for the negative control well (CD4^+^ T cells alone and conditional medium, separately) and the positive control well (CD4^+^ T cells with 5 ng/ml rhTGFβ1) were also performed. Spots were developed with human IL-10 detection antibody, streptavidin-horseradish peroxidase, and 3-amino-9-ethylcarbazole substrate set (BD Biosciences, Cat. #551951). The spot-forming cells were imaged and enumerated in each experimental well.

### Enzyme-linked immunosorbent assay

KYSE30 *ITGAX-*OE/vector and KYSE150 *ITGAX*-OE/vector cells were seeded in a 6-well plate at a density of 5.0 × 10^5^ cells per well. After 48 h of incubation, the cell supernatants were centrifuged at 500 ×*g* for 5 min, and the supernatants were collected. The protein level of TGFβ1 was analyzed using a human TGFβ1 enzyme-linked immunosorbent assay (ELISA) kit (Proteintech, Cat. #KE00002) according to the manufacturer’s instructions. Data from at least 3 independent experiments in triplicate were presented.

### In vitro cell phenotype assay

We seeded ESCC cells in 96-well plates and assessed cell viability at 5 time points using Cell Counting Kit-8 (DOJINDO, Cat. #CK04). Cells in exponential growth were harvested and resuspended to a single-cell suspension of 1.0 × 10^3^/ml cells with complete medium. After incubation for 2 weeks at 37 °C under 5% CO_2_ in a humidified incubator, the colonies were fixed with methanol, stained with crystal violet, and subsequently imaged and quantified. Invasion assays were conducted using Millicell chambers coated with 80 μl of Matrigel (500 ng/μl). Cells were seeded onto the coated filters in serum-free medium and incubated for 18 to 22 h. The migrated cells were subsequently fixed with methanol and stained with crystal violet. The migration assays were performed in a similar manner, but without the Matrigel coating on the filters. All assays were performed 3 times, with 3 replications per assay.

### Mouse tumor xenograft growth and metastasis assays

For examination of ESCC xenograft growth in mice, KYSE150 cells (2.0 × 10^6^) with or without *ITGAX* stable overexpression or knockout were suspended in 100 μl of PBS and injected into the hind flank of 6-week-old NSG mice (*n* = 5 per group). We palpated tumor development and monitored the growth rate by measuring the tumor volume with a caliper every 3 days. Tumor volume was determined by length × width^2^ × 0.52.

For examination of ESCC cell metastasis in mice, KYSE150 cells (2.5 × 10^5^) with or without *ITGAX* stable overexpression were suspended in 50 μl of PBS and injected into 6-week-old BALB/c mice through the tail vein (*n* = 7 per group). The mice were monitored for general health status and evidence of morbidity related to metastasis. After inoculation for 8 weeks, the mouse lungs were removed, fixed, and embedded in paraffin for further analysis.

For humanized mouse model establishment and immune checkpoint inhibitor (ICI) therapy, KYSE150 cells (2.5 × 10^5^) with or without *ITGAX* stable overexpression were harvested and resuspended in 50 μl of PBS per mouse, followed by tail vein injection into NSG mice. On day 7 of injection, mice were randomly divided into 4 groups (*n* = 5 per group) and injected with human PBMCs (5 × 10^6^) isolated from healthy donors and resuspended in 50 μl PBS, or an equal volume of PBS as vehicle. To examine the therapeutic effect of ICI or ICI plus SIS3 (Sellerk, Cat. #S3552), humanized mice were treated with a human anti-PD-L1 monoclonal antibody (Sellerk, Cat. #A2004) weekly at 10 mg per kg body weight via intraperitoneal injections for a total of 4 doses since the day human PBMCs were transferred. To check the efficacy of PD-L1 antibody plus SIS3, mice were administered intraperitoneally with SIS3 dissolved in 2% DMSO, 40% PEG300, 2% Tween-80, and 56% PBS at 5 mg/kg every 2 days for 12 times. Mice in the control group were administered with the same amount of solvent. Mice were weighed, and tumor volumes were measured every 2 days and sacrificed 7 days after therapy completion (day 35). The lungs removed were processed for histopathological examination and tumor-infiltrating lymphocyte analysis by flow cytometry.

### Real-time quantitative PCR

Total RNA was obtained with RNA-Quick Purification Kit (ES Science, Cat. #RN001), and cDNA was synthesized from RNA using a reverse transcription kit (Takara, Cat. #RR037A). Real-time PCR was performed using Power SYBR Green RT-PCR Reagent (Takara, Cat. #RR420A). The PCR primer sequences are shown in Table S2. All samples were assayed in triplicate, with *GAPDH* as the endogenous reference.

### Immunoblot analysis

The antibodies used for immunoblotting analysis are shown in Table S1. We extracted total protein from tissues or cells using RIPA lysis buffer (Beyotime, Cat. #P0013D) containing Protease and Phosphatase Inhibitor Cocktail (NCM Biotech, Cat. #P002). Total protein separated by sodium dodecyl sulfate–polyacrylamide gel electrophoresis (Vazyme, Cat. #E306-01) was transferred to a polyvinylidene fluoride membrane (Millipore, Cat. #IPVH00010) and then incubated with a primary antibody, followed by incubation with a secondary antibody (Cell Signaling Technology). The protein signals were detected in Amersham Imager 600 using a high-sensitivity ECL chemiluminescence detection kit (Vazyme, Cat. #E412).

### Immunoprecipitation assay

Total proteins were extracted from cells using a weak RIPA lysis buffer (Beyotime, Cat. #P0013D) containing a Protease and Phosphatase Inhibitor Cocktail (NCM Biotech, Cat. #P002). We conducted immunoprecipitation with CD11c or SMAD3 antibody using Pierce Classic Magnetic IP/Co-IP Kit (Invitrogen, Cat. #88804). Protein lysates (1 mg) were incubated with primary antibodies anti-SMAD3, anti-CD11c, anti-FLAG, anti-mouse IgG (5 μg per immunoprecipitation), or anti-rabbit IgG (5 μg per immunoprecipitation) overnight at 4 °C (Table S1). The protein–antibody complexes were then incubated with 25 μl of Protein A/G Magnetic Beads for 2 h. Beads were then washed 4 times with 1 ml of Wash Buffer, followed by elution. The eluted products were then immunoblotted. Secondary antibody against IgG light chain was obtained from Abbkine (Cat. #A25022).

### Immunofluorescence assay

KYSE30 and KYSE150 cells were grown to 50% to 70% confluency on coverslips (Solarbio). After PBS washing, cells on coverslips were fixed with 4% paraformaldehyde and permeabilized with 0.2% Triton X-100. The coverslips were blocked with 3% bovine serum albumin and then incubated with antibodies against CD11c, phosphorylated SMAD3 (p-SMAD3), and TGFBR1 (Table S1). Goat anti-mouse secondary antibody with Alexa Fluor 594, goat anti-mouse secondary antibody with Alexa Fluor 633, and goat anti-rabbit secondary antibody with Alexa Fluor 488 from Thermo Fisher were used as the secondary antibody. Confocal microscopy was used to capture the images after coverslips were sealed with a mounting medium containing DAPI (ZSGB-BIO, Cat. #ZLI-9557). To investigate the localization of exogenous SMAD3, cells were labeled with the membrane-specific red fluorescent dye DiI (Beyotime, Cat. #C1036).

### Chromatin immunoprecipitation-coupled quantitative PCR analysis

We used SimpleChIP Plus Sonication Chromatin IP Kit (Cell Signaling Technology, Cat. #56383) to carry out chromatin immunoprecipitation. Human esophageal squamous cell HET-1A and ESCC cells (KYSE30 and KYSE150) were fixed and cross-linked with formaldehyde before immunoprecipitation with p-SMAD3 antibody (Cell Signaling Technology, RRID: AB_2193207) or rabbit IgG antibody (Cell Signaling Technology, Cat. #2729). DNA fragments were quantified using qPCR with the primers shown in Table S2.

### Fluorescence in situ hybridization

*ITGAX* gene copy number variation was assessed by fluorescence in situ hybridization (FISH) in HET-1A, KYSE150, and KYSE30 cells. Probes against the *ITGAX* loci were designed using OligoMiner and subjected to BLASTn (https://blast.ncbi.nlm.nih.gov/Blast.cgi) searches to eliminate nonspecific final probe sets. The final probes consisted of 15 probes and were tagged with the 5′ Cy3 fluorophore. Briefly, cells on the coverslips were fixed with 4% paraformaldehyde for 20 min at room temperature and washed twice with diethyl pyrocarbonate (DEPC)-treated water. Coverslips were heated in denaturation buffer (70% formamide, 2× saline sodium citrate [SSC]) at 75 °C for 8 min and dehydrated sequentially with 70%, 80%, 90%, and 100% ethanol for 5 min. In total, 20 μl of *ITGAX* probe was applied per slide and incubated at 37 °C for 16 h. Coverslips were washed with 2× SSC at 53 °C for 5 min, then with 2× SSC containing 0.1% NP-40 at 42 °C for 5 min, followed by 2× SSC at 42 °C for 5 min. Coverslips were then counterstained with DAPI, and fluorescence images were captured using high-resolution confocal microscopy. Signal thresholds were manually set to remove background fluorescence, and FISH signals were quantified using ImageJ (v1.8.0).

### Public omics’ datasets

Origins of public omics’ datasets are noted when used in the main text and are listed as follows: Multistage esophageal tissue samples’ whole exome sequencing (WES) and whole genome sequencing (WGS) data [25] are deposited in GSA-Human (Genome Sequence Archive in BIG Data Center, Beijing Institute of Genomics, Chinese Academy of Sciences, https://bigd.big.ac.cn/gsa-human) as PRJCA015964. Multistage esophageal tissue samples’ single-cell RNA sequencing (scRNA-seq) and spatial RNA-seq data [14] were accessed under HRA000776 in GSA-Human. Multistage esophageal tissue samples’ proteome and phosphoproteome data [26] were reached through PXD038961 in the ProteomeXchange Consortium. ESCC bulk RNA-seq data [16] are available in the Gene Expression Omnibus (GEO) database with accession number GSE160269. The scRNA-seq data of pretreatment ESCC patients [27] is deposited in GSA-Human under accession number HRA003591. TCGA data, which support the findings of this study, are available from the TCGA database (http://cancergenome.nih.gov) and TumorPortal (http://www.tumorportal.org). All remaining data are available in the article and the Supplementary Materials. Any additional information or code required to reanalyze the data reported in this study is available upon request.

### Statistics

We performed statistical analyses using R 4.1.3 and GraphPad Prism 9. The corresponding statistical methods and details are described in the figure legends, text, or specific methods. *P* values were computed using the Wilcoxon rank-sum test or Student *t* test. Otherwise, the methods could be noted in the corresponding figure legends. Values from at least 3 independent assays are presented as mean ± standard error of the mean (SEM). Values from ≥3 biological replicates are presented as mean ± standard deviation (SD). Error bars represent the mean ± SD or SEM, as specified in the corresponding figure legends.

## Results

### Revealing CD11c-positive epithelial cell subtype in ESCC development

**Fig. 1.**
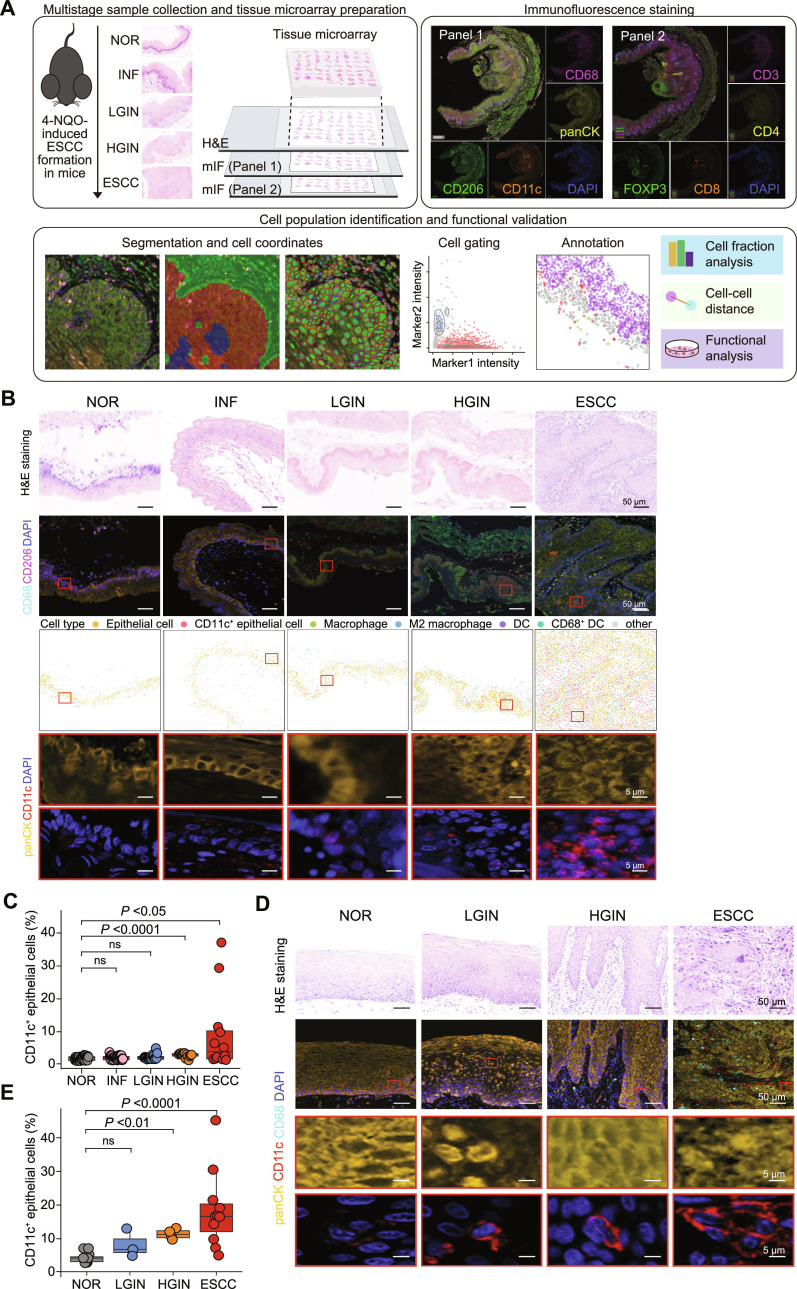
Revealing CD11c-positive epithelial cell subtype in ESCC development. (A) Overall workflow of mIF detection of multistage esophageal precancerous and cancerous lesion samples from 4-NQO-treated mice. Panels show multistage sample collection, immunofluorescence staining, cell identification, and functional examination. (B) Representative mIF images of tissue sections with identified cell phenotypes. Panels from top to bottom are H&E images inferred by inForm software, mIF staining images at the same location, cells annotated with different subtypes based on fluorescence intensity thresholds determined by Otsu’s method, and magnified images of CD11c and panCK immunofluorescence images in the ROIs in multistage tissues. mIF staining markers are shown on the left side of panels, panCK (gold), CD11c (red), CD68 (cyan), CD206 (magenta), and DAPI (blue, for cell nuclei). In the third row of the panel, dots in different colors represent cell types, and the colors are denoted above the panel. (C) Fraction of CD11c^+^ epithelial cells in different histopathological stages of esophageal carcinogenesis in 4-NQO-treated mice. Sample size at each stage: NOR, 30; INF, 37; LGIN, 20; HGIN, 14; ESCC, 12. (D) Representative CD11c mIF images of human esophageal tissues at different pathological stages. Panels from top to bottom are H&E staining, mIF staining of relative serial sections, and magnified images of CD11c, CD68, and panCK in the ROIs in multistage tissues. CD11c (red), CD68 (cyan), panCK (gold), and DAPI (blue, for cell nuclei). (E) Fraction of CD11c^+^ esophageal epithelium in different histopathological stages of human ESCC development (NOR, *n* = 12; LGIN, *n* = 3; HGIN, *n* = 4; ESCC, *n* = 11). *P* values were calculated from the Wilcoxon rank-sum test (C and E). ns, not significant. Abbreviations: 4-NQO, 4-nitroquinoline 1-oxide; NOR, normal epithelial tissue; INF, inflammatory tissue; LGIN, low-grade intraepithelial neoplasia tissue; HGIN, high-grade intraepithelial neoplasia tissue; ESCC, esophageal squamous cell carcinoma; H&E, hematoxylin and eosin; mIF, multiplex immunofluorescence; DC, dendritic cell; ROI, regions of interest.

First, we conducted mIF assays on TMAs containing 123 mouse esophageal tissue samples with multistage precancerous lesions or ESCC, including 52 NOR, 27 INF, 21 LGIN, 10 HGIN, and 13 ESCC samples, using 2 antibody panels targeting epithelial cells, T cells, or myeloid cells (Fig. S1A). Epithelial cells were identified using panCK; T cell subtypes were identified using CD3, CD4, CD8, and FOXP3; and myeloid cells were identified using CD68, CD206, and CD11c (Fig. 1A). After manual evaluation of the robustness and specificity of mIF staining, we used InForm software to detect the epithelium–stroma boundary and conduct cell segmentation. Consequently, 317,801 cells from Panel 1 and 301,385 cells from Panel 2 passed quality control (Table S3). The Otsu classification algorithm was applied to normalize the background fluorescence intensity of each slide, set positive thresholds for each marker, and identify cell phenotypes for each individual sample based on the effective marker intensity (positive/negative; Fig. 1A). Specifically, we used panCK to represent the distribution of epithelial cells, owing to its epidermal keratin specificity. Within the panCK^-^ nonepithelial cell population, CD11c^+^ dendritic cells (DCs) were divided into CD68^−^ and CD68^+^ subtypes, and macrophages identified by CD68 were further categorized into M2 or other subtypes based on CD206 expression (Fig. 1B).

This approach revealed a unique cluster of CD11c^+^ cells among the panCK^+^ epithelial population (Fig. 1B), a notable discovery because CD11c (encoded by the *ITGAX* gene) is a classical marker for professional antigen-presenting cells (APCs), which are typically associated with immune cells [28,29]. Notably, we observed an increase in the number of CD11c^+^ epithelial cells across the stepwise malignant transformation (Fig. 1C). M2 macrophages, which are known to promote immunosuppressive microenvironment, were located closer to CD11c^+^ epithelial cells in ESCC than in other earlier precancerous stages via cell–cell distance analysis (Fig. S1B). More CD11c^+^ tumor cells were found in proximity to M2 macrophages in ESCC than in other precancerous stages (Fig. S1C), suggesting that CD11c^+^ epithelial cells may be associated with the establishment of an immunosuppressive niche in ESCC. Therefore, we further validated the presence of CD11c^+^ epithelial cell clusters. By performing flow cytometry on normal mouse esophageal epithelium and ESCC samples, we confirmed that CD11c^+^ epithelia existed and expanded in ESCC initiation, with a proportion from 2.44% (range, 2.13% to 2.65%; *n* = 4) in normal esophageal epithelia to 28.63% (range, 23.10% to 32.60%; *n* = 3) in ESCC (Fig. S1D and E). Our findings were not limited to mouse models. We extended our analysis to human samples and confirmed the presence of CD11c^+^ epithelial cells in human esophageal tumorigenesis, with their proportion gradually increasing as the disease progressed (Fig. 1D and E) and further validated the expression in ESCC cell lines (Fig. S1F). These results indicate that CD11c^+^ epithelial cells may contribute to ESCC formation in both mouse and human and may be associated with immunomodulation.

### CD11c expression in esophageal epithelial cells promoted ESCC formation and progression

**Fig. 2.**
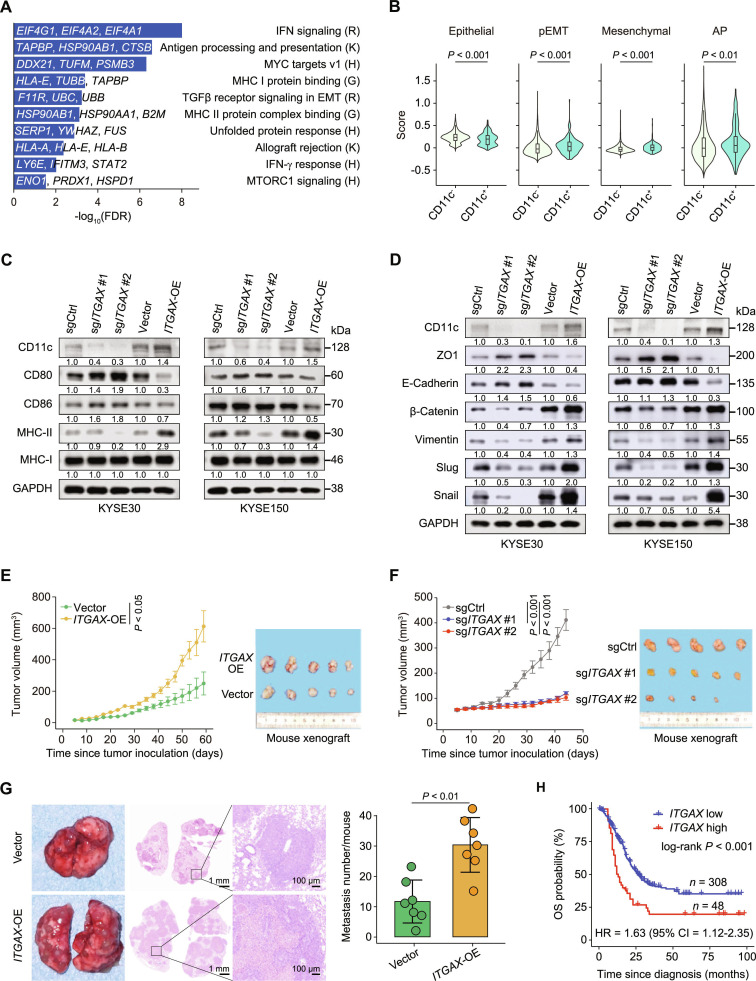
CD11c expression in esophageal epithelial cells promotes ESCC formation and progression. (A) Pathway enrichment for genes differentially expressed in CD11c^+^ versus CD11c^−^ human ESCC epithelial cells identified by spatial transcriptomics analysis [14]. Significant pathways (FDR < 0.05) and genes up-regulated in CD11c^+^ epithelial spots are shown. Pathway databases: R, Reactome; K, KEGG; H, Hallmarks; G, Gene Ontology. (B) Violin plots displaying the scaled epithelial program scores of epithelial cells divided by CD11c expression status derived from scRNA-seq data [16]. (C and D) Immunoblot of AP pathway (C) and EMT pathway (D) markers in *ITGAX* KO or OE KYSE30 and KYSE150 cells. Each experiment had 3 independent repeats. (E and F) Effect of *ITGAX*- OE (E) or KO (F) on the growth of mouse subcutaneous xenografts of KYSE150 cells. *n* = 5 mice per group. Left, tumor size at different time points. Right, representative image of tumor resected at the last experimental time point from the left panel (day 59 in E and day 44 in F). (G) Effect of *ITGAX*-OE on lung colonization of KYSE150 cells in mice. Leftmost, representative gross images of lung metastases. Middle, H&E staining, and magnified ROI regions. Rightmost, quantitative statistics of lung metastases. Data are presented as mean ± SD from 7 mice. (H) Kaplan–Meier estimate of overall survival time in 356 ESCC patients grouped by *ITGAX* mRNA level (cutoff decided by survminer R package, see the “Patient survival analysis” section), and the significance *P* value was obtained by the log-rank test. HR was calculated by multivariate Cox proportional hazard models with age, sex, and tumor stage as covariates. *P* values were from the Wilcoxon rank-sum test (B and G) or the Student *t* test (E and F). Abbreviations: FDR, false discovery rate; pEMT, partial EMT; AP, antigen presenting; *ITGAX*, integrin alpha X; sgCtrl, single guide RNA control; sg*ITGAX*, single guide RNA targeting *ITGAX* gene; Vector, control for OE group; OE, overexpression; MHC-II, MHC-II proteins; MHC-I, MHC-I proteins; ZO1, tight junction protein ZO-1, related to EMT; E-Cadherin, protein for *CDH1*; β-Catenin, protein for *CTNNB1*; Vimentin, protein for *VIM*; Slug, protein for *SNAI2*; Snail, protein for *SNAI1*; OS, overall survival; HR, hazard ratio; CI, confidence interval; ROI, regions of interest.

To explore the functional role of CD11c^+^ epithelial cells in ESCC progression, we utilized previously reported spatial transcriptomic data of human multistage esophageal samples from 4 patients with ESCC [14], with clearly identifiable epithelium identities, to compare CD11c^+^ with CD11c^−^ cells (defined as cells expressing *ITGAX* mRNA or not). Results revealed that CD11c^+^ epithelial spots increased as the disease progressed (NOR/INF, 7.58%; LGIN, 12.75%; HGIN, 11.11%; ESCC, 13.28%; *P* value for trend in proportions was <0.001 [chi-squared test]), consistent with mouse and human mIF findings. Further comparison of 769 CD11c^+^ and 6,943 CD11c^−^ epithelial spots revealed 86 up-regulated genes in CD11c^+^ versus CD11c^−^ epithelial cells (fold change > 2, FDR < 0.05). These differentially overexpressed genes in CD11c^+^ cells were enriched in multiple pathways, including IFN-γ signaling, antigen processing and presentation, MYC targets v1, major histocompatibility complex (MHC) class I protein binding, TGFβ receptor signaling in EMT, and MHC class II protein complex binding (Fig. 2A and Table S4). Notably, immune-related pathways such as IFN-γ response, antigen processing and presentation, together with the EMT-related TGFβ receptor signaling pathways, suggest that CD11c^+^ epithelial cells may play a dual role in immunomodulatory and promoting EMT in ESCC. Single-cell transcriptome data from human ESCC [16] confirmed this functional characterization. By estimating the mean expression score based on representative gene panels from public literature [16,30,31], we demonstrated that CD11c^+^ epithelial cells had a significantly lower epithelial score but significantly higher partial EMT, mesenchymal, and AP scores compared to CD11c^−^ epithelial cells (Fig. 2B and Table S5).

We then sought to confirm the in vitro functional impact of CD11c on the ESCC AP and EMT pathways by conducting *ITGAX* knockout (KO) or overexpression (OE) in the ESCC cell lines KYSE30 and KYSE150. MHC class I genes human leukocyte antigen (*HLA*)*-A*, *HLA-B*, and *B2M* (beta-2-microglobulin); MHC class II genes *HLA-DRA*, *HLA-DQA1*, and *HLA-DPA1* (DR, DQ, and DP α chains, respectively); and costimulatory molecules *CD80* and *CD86* were selected as representative markers of the AP pathway, given their crucial roles in the antigen processing machinery. The results showed that *ITGAX*-KO increased the expression of *CD80* and *CD86* while reducing MHC class II gene expression. In contrast, *ITGAX-*OE markedly increased the expression of MHC class II genes but suppressed *CD80* and *CD86* expression without affecting MHC class I genes (Fig. 2C and Fig. S2A and B). Additionally, *ITGAX-*KO down-regulated the mesenchymal-related genes (*CDH2* [cadherin-2], *SNAI1* [snail family transcriptional repressor 1], *SNAI2* [snail family transcriptional repressor 2]*,* and *VIM* [vimentin]), but up-regulated the epithelial-related gene *CDH1* (cadherin-1) at both the mRNA and protein levels, whereas *ITGAX*-OE had the opposite effect (Fig. 2D and Fig. S2C). These findings suggest that CD11c may disrupt MHC class II-mediated AP [32] and induce EMT in human ESCC.

Moreover, CD11c-driven malignant progression in ESCC highlights the clinical significance of this protein. *ITGAX*-KO significantly suppressed, whereas *ITGAX-*OE significantly enhanced ESCC cell proliferation, migration, and invasion in vitro (Fig. S2D and E). This was further confirmed in mouse subcutaneous xenografts and in a lung metastatic model using KYSE150 cells, wherein *ITGAX*-OE significantly promoted ESCC cell proliferation and metastasis (Fig. 2E to G). Finally, we evaluated the clinical value of *ITGAX* in a human ESCC cohort of 356 patients (combination of 4 external cohorts). Results revealed that a high *ITGAX* mRNA level was related with poor overall survival in patients with ESCC (log-rank *P* < 0.001), with an HR adjusted for sex, age, and tumor stage of 1.63 (95% confidence interval, 1.12 to 2.35; Fig. 2H). This clinical relevance highlights the potential of CD11c expression as a prognostic biomarker and therapeutic target; however, further validation in the context of CD11c^+^ epithelial cells is required.

### CD11c expression in epithelial cells induces suppressive AP process and EMT via SMAD3

**Fig. 3.**
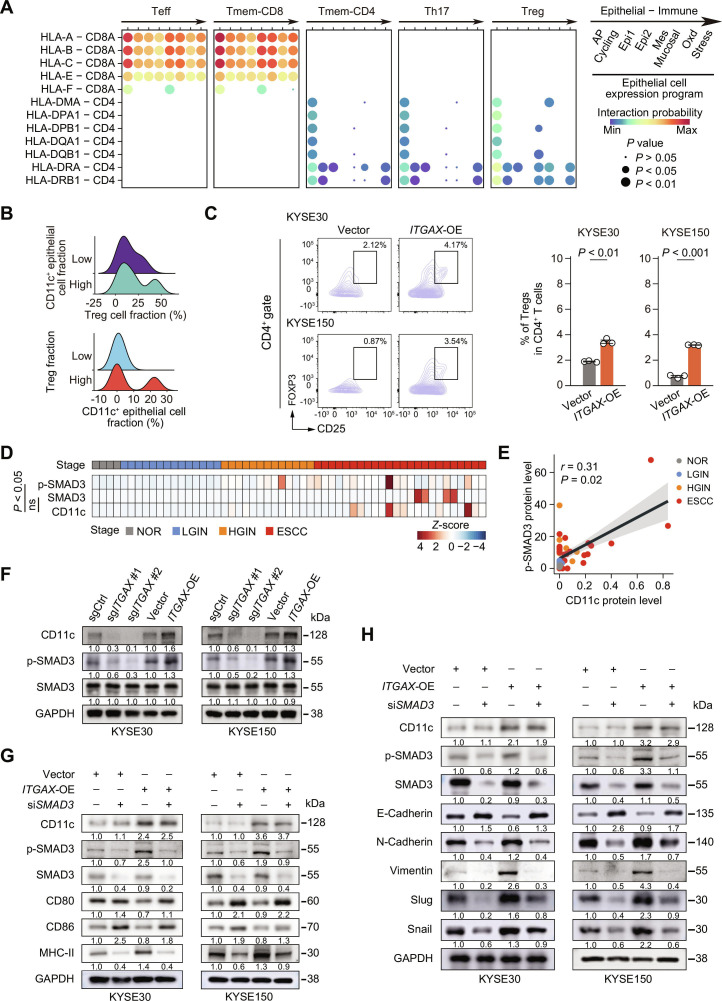
Epithelial CD11c expression induces a suppressive antigen-presenting process and EMT via SMAD3. (A) Bubble plots depicting the interactions between various epithelial cell programs and selected T cell subtypes based on scRNA-seq data. The dot size indicates the *P* value from the permutation test. Color indicates the average interaction probability of the receptor–ligand pairs between each of the 2 clusters. MHC class I-related interactions are represented by HLA-A, HLA-B, HLA-C, HLA-E, and HLA-F interacting with CD8A, whereas MHC class II-related interactions are represented by HLA-DMA, HLA-DPA1, HLA-DPB1, HLA-DQA1, HLA-DQB1, HLA-DRA, and HLA-DRB1 interacting with CD4. (B) Ridgeline plot showing the distribution of Tregs in mouse ESCC tissues, based on CD11c^+^ epithelial fraction (upper panel, samples were stratified into high and low CD11c^+^ epithelial cell fraction groups using median value based on mIF quantification) and the distribution of CD11c^+^ epithelium with different Treg infiltrations in mouse ESCC tissues (lower panel, samples were stratified into high and low Treg cell fraction using median value based on mIF quantification). (C) Effect of *ITGAX*-OE in KYSE30 and KYSE150 cells on Treg cell induction in cocultured human naive CD4^+^ T cells, as determined by flow cytometry. Left, examples of Treg cell identification from flow cytometry. Right, quantification of Treg proportions in CD4^+^ T cells. (D) Heatmap of scaled p-SMAD3, SMAD3, and CD11c protein levels in different human histopathological esophageal tissues from patients with available data (*n* = 55) [26]. *P* value and “ns” denote statistical significance based on Spearman correlation analysis. (E) Spearman correlation between CD11c and p-SMAD3 protein levels in different histopathological tissues of the human esophagus [26], related to (D). (F) Immunoblot analysis of p-SMAD3 and total SMAD3 in *ITGAX*-KO or *ITGAX*-OE KYSE30 and KYSE150 cells. (G and H) Effect of *SMAD3* knockdown (si*SMAD3*) on MHC class II-mediated AP (G) and EMT (H) in KYSE30 and KYSE150 cells with or without *ITGAX*-OE. Each immunoblot had 3 independent repeats. Abbreviations: AP, antigen presentation; Cycling, cell cycle; Epi, epithelial expression program; Mes, mesenchymal-like; Mucosal, mucosal defense; Oxd, oxidative stress or detoxification; Stress, stress responses; Teff, effective T cells. Tmem-CD8, memory CD8 T cells; Tmem-CD4, memory CD4 T cells; Th17, T helper 17 cells; Treg, regulatory T cells; NOR, normal epithelial tissue; LGIN, low-grade intraepithelial neoplasia tissue; HGIN, high-grade intraepithelial neoplasia tissue; ESCC, esophageal squamous cell carcinoma; *ITGAX*, integrin alpha X; sgCtrl, single guide RNA control; sg*ITGAX*, single guide RNA targeting *ITGAX* gene; Vector, control for OE group; OE, overexpression; SMAD3, mothers against decapentaplegic homolog 3; p-SMAD3, phosphorylated SMAD3; MHC-II, MHC-II proteins; MHC-I, MHC-I proteins; ZO1, tight junction protein ZO-1, related to EMT; E-Cadherin, protein for *CDH1*; N-Cadherin, protein for *CLDN2*; Vimentin, protein for *VIM*; Slug, protein for *SNAI2*; Snail, protein for *SNAI1*.

Although high MHC class II levels indicate the maturation of professional APCs, the lack of costimulatory molecules (CD80 and CD86) causes immune dysfunction, particularly through MHC class II-dependent interactions with T cells [33]. We hypothesized that while high MHC class II expression was observed in CD11c-overexpressing ESCC cells, the concurrent down-regulation of CD80/CD86 suggested nonproductive AP. To test this, we explored epithelial–T cell interactions using scRNA-seq data from human ESCC [16] and found that MHC class II in AP epithelial cells had strong interactions with CD4^+^ suppressive T cells (Tregs; Fig. 3A). Meanwhile, joint mIF analysis of the 2 panels revealed a trend toward a higher proportion of Tregs in tissues with more CD11c^+^ epithelial cells than in those with a low CD11c^+^ epithelial cell fraction, and vice versa, although the statistical comparison between groups did not reach the level of significance (average Treg fraction: 12.8% in samples with CD11c^+^ epithelium vs. 11.7% in samples with CD11c^−^ epithelium; *P* = 0.95, Wilcoxon rank-sum test) (Fig. 3B). These findings suggest that MHC class II-mediated AP in epithelial cells may actively engage in suppressive CD4^+^ T cell responses, and that CD11c may assist in this process. To further assess the immunosuppressive function of CD11c^+^ epithelial cells, we performed coculture experiments with human CD4^+^ T cells. Flow cytometry assays showed that there were significantly more Tregs and immunosuppressive cytokine IL-10-secreting cells when CD4^+^ T cells were cocultured with *ITGAX-*OE ESCC cells compared with those cocultured with Vector-control ESCC cells, while the proportion of IFN-γ^+^CD4^+^ T cells was significantly decreased in coculture with *ITGAX-*OE ESCC cells compared with those cocultured with control ESCC cells (Fig. 3C and Fig. S3A), indicating that *ITGAX-*OE ESCC cells reduced the anti-tumor activity of CD4^+^ T cells. Additionally, ELISpot assays revealed that ESCC promoted CD4^+^ T cells to secrete the immunosuppressive cytokine IL-10. The number of IL-10 spots significantly increased under *ITGAX*-OE ESCC cell coculture, to the degree of TGFβ1 stimulation, indicating a tolerogenic interaction (Fig. S3B). These findings highlight a tumor-intrinsic immunomodulatory AP process of CD11c^+^ epithelial cells, potentially contributing to immune evasion, wherein the down-regulation of CD80/CD86 induces immunosuppressive CD4^+^ T cell activation.

We then explored the molecular mechanisms underlying the dual effects of epithelial CD11c on AP and EMT activation, as previous evidence suggests common regulatory pathways, such as TGFβ (Fig. 2A) [8]. Our transcriptomic inspection revealed no significant correlation between the expression of CD11c and core components of the TGFβ family genes. ELISA also confirmed that CD11c level did not affect TGFβ1 secretion level (Fig. S3C and D). Notably, by examining a publicly available proteomic dataset from multistage human ESCC samples [26], we discovered that CD11c expression was positively correlated with p-SMAD3 levels, instead of total SMAD3 levels, in epithelial cells during ESCC development (Fig. 3D and E). Correspondingly, *ITGAX*-KO substantially decreased while OE increased p-SMAD3 levels with no effect on total SMAD3 (Fig. 3F). To examine whether SMAD3 is pivotal for CD11c-triggered AP and EMT, we knocked down *SMAD3* in *ITGAX*-OE ESCC cell lines. Changes in *ITGAX* and *SMAD3* expression did not alter expression levels of the key TGFβ pathway genes including *SMAD2*, *TGFBR1,* and *TGFBR2* (Fig. S4A); however, knockdown of *SMAD3* significantly suppressed the levels of key MHC class II genes (*HLA-DRA*, *HLA-DQA1*, and *HLA-DPA1*; Fig. 3G and Fig. S4B) and EMT markers (*CDH2*, *SNAI1*, and *SNAI2*; Fig. 3H and Fig. S4C) at both the mRNA and protein levels, as well as migration and invasion phenotypes (Fig. S4D), irrespective of *ITGAX* status. Additionally, the expression levels of *CD80, CD86*, and *CDH1* were significantly elevated when *SMAD3* was knocked down (Fig. 3G and H and Fig. S4B and C), suggesting that CD11c triggers EMT and elicits immunosuppressive AP through p-SMAD3, which is a dual effector involved in both pathways.

To further confirm the role of *ITGAX* and *SMAD3* expression, we performed in vivo p-SMAD3 rescue experiments using SIS3, a selective SMAD3 phosphorylation inhibitor. It turned out that SIS3 profoundly suppressed the growth of mouse subcutaneous xenografts (Fig. S5A). We also generated an NSG mouse model where *ITGAX*-OE or Vector-control KYSE150 cells were injected on day 0, SIS3 or its vehicle was administered from day 7 to day 28 every other day, and lung metastases were collected on day 35 (Fig. S5B). We observed that SIS3 significantly inhibited the lung metastasis of tumors derived from *ITGAX*-OE ESCC cells (Fig. S5C). Meanwhile, mIF analysis of the lung metastases showed that treatment of SIS3 significantly reduced the p-SMAD3 levels, but the myeloid components (panCK^-^CD11b^+^F4/80^+^ for macrophages and panCK^-^CD11b^+^CD11c^+^ for DCs) were not influenced by CD11c and p-SMAD3 levels (Fig. S5D and E). Together, these results indicate that p-SMAD3 is the key molecule for epithelial CD11c function.

### CD11c mediates SMAD3 phosphorylation and nuclear translocation for immune modulation

**Fig. 4.**
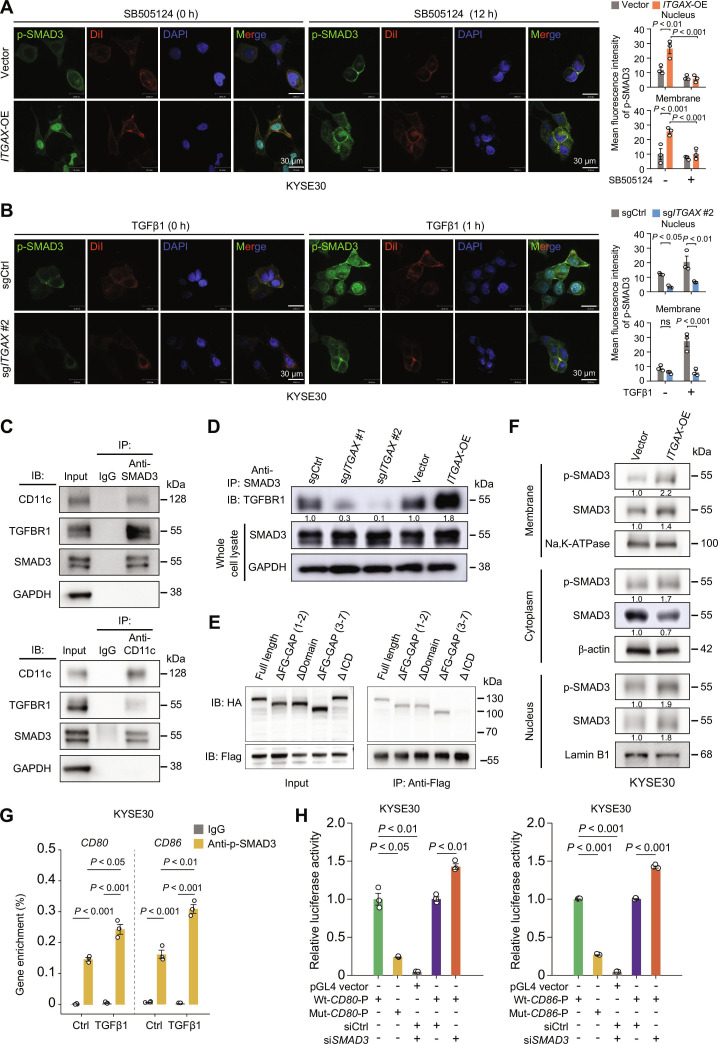
CD11c mediates phosphorylated SMAD3 nuclear translocation to perform its functions. (A) Left panel, representative confocal images showing p-SMAD3 levels in *ITGAX-*OE KYSE30 cells treated without or with TGFBR1 inhibitor SB505124. Antibody for each channel is labeled in the panels: p-SMAD3 (green), DiI (red, for cell membrane), and DAPI (blue, for cell nuclei). Right panel, quantitative results of mean fluorescence intensity of p-SMAD3. Data represent mean ± SEM from 3 independent experiments. (B) Left panel, representative confocal images showing *ITGAX-*KO (sg*ITGAX*) KYSE30 cells treated without or with TGFβ1. Antibody for each channel is labeled in the panels: p-SMAD3 (green), DiI (red, for cell membrane), and DAPI (blue, for cell nuclei). Right panel, quantitative results of mean fluorescence intensity of p-SMAD3. Data represent mean ± SEM from 3 independent experiments. (C) Immunoblot of the coimmunoprecipitation products collected from whole cell lysates with SMAD3 (upper panel) or CD11c antibody (lower panel) in KYSE30 cells. (D) Western blot analysis of immunoprecipitation products collected using SMAD3 antibody shows the interaction of TGFBR1-SMAD3 in KYSE30 cells with *ITGAX* KO or OE. (E) Coimmunoprecipitation assay of the binding of CD11c truncated mutants and SMAD3 (Flag). (F) Cell membrane (marked by Na, K-ATPase), cytoplasm (marked by β-actin), and nucleus (marked by Lamin B1) fractions of *ITGAX*-OE KYSE30 cells were collected and subjected to IB analysis of p-SMAD3 and SMAD3. (G) Chromatin immunoprecipitation-coupled qPCR assays show enrichment of *CD80* or *CD86* promoter in cell lysates obtained with anti-p-SMAD3 antibody in KYSE30 cells stimulated with vehicle (Ctrl) or TGFβ1. (H) Luciferase reporter assays in KYSE30 cells using the indicated reporter plasmids of *CD80* (left) or *CD86* (right) promoters or siRNA targeting *SMAD3*. Data are mean ± SEM from 3 experiments, and each had 3 replicates. *P* values from Student *t* test. Abbreviations: *ITGAX*, integrin alpha X; sgCtrl, single guide RNA control; sg*ITGAX*, single guide RNA targeting *ITGAX* gene; Vector, control for OE group; OE, overexpression; Δ, deletion; FG-GAP (1 and 2), Phenylalanine-Glycine-GAP repeat fragment 1 to 2; FG-GAP (3 to 7), Phenylalanine-Glycine-GAP repeat fragment 3 to 7; ICD, intracellular domain; Wt-*CD80*-P, wild-type *CD80* promoter; Mut*-CD80*-P, mutant-type *CD80* promoter (without p-SMAD3 binding motif); Wt-*CD86*-P, wild-type *CD86* promoter; Mut-*CD86*-P, mutant-type *CD86* promoter (without p-SMAD3 binding motif); SMAD3, mothers against decapentaplegic homolog 3; p-SMAD3, phosphorylated SMAD3;. HA, HA tag, CD11c-Δ; Flag, Flag-SMAD3; TGFBR1, transforming growth factor beta receptor I; IB, immunoblot; IP, immunoprecipitation; TGFβ1, transforming growth factor beta 1; Ctrl, control.

SMAD3 plays a key role in the TGF pathway, which requires phosphorylation by TGFBR1 on the plasma membrane. After SMAD3 phosphorylation, the SMAD2/SMAD3 complex separates from TGFBR1 and translocates to the nucleus to regulate downstream regulons [34]. To elucidate how CD11c affects p-SMAD3 expression in ESCC cells, we examined the subcellular localizations of SMAD3, TGFBR1, and CD11c. IHC revealed a spontaneous colocalization of these 3 proteins in the cellular membrane and a strong nuclear signal of SMAD3 in CD11c^+^ esophageal epithelial cell line HET-1A and ESCC cells (KYSE30 and KYSE150) upon TGFβ1 stimulation (Fig. S6A). To further investigate the role of CD11c in p-SMAD3 distribution, we assessed the subcellular distribution of p-SMAD3 in ESCC cell lines with either *ITGAX-*OE or KO. Compared to the control, *ITGAX-*OE cells exhibited elevated endogenous p-SMAD3 protein levels with increased nuclear distribution, which was effectively suppressed by the TGFBR1 inhibitor SB505124 (Fig. 4A and Fig. S6B). Conversely, compared to sgCtrl cells, *ITGAX*-KO cells exhibited decreased p-SMAD3 levels and diminished nuclear translocation of p-SMAD3 (Fig. 4B and Fig. S6C). Additionally, coimmunoprecipitation assays confirmed that endogenous CD11c interacted with SMAD3 in ESCC cells (Fig. 4C and Fig. S7A). *ITGAX*-OE substantially increased the interaction between SMAD3 and its phosphorylating activator, TGFBR1 (Fig. 4D and Fig. S7B). We then generated HA-tagged truncation mutants of CD11c and Flag-tagged full-length SMAD3 and transfected them into HEK-293T cells (Fig. S7C). We found that deletion of the intracellular domain markedly impaired its binding to SMAD3, while the extracellular domain was dispensable (Fig. 4E). Subcellular fractionation assays of control or *ITGAX-*OE ESCC cells (Fig. S7D) demonstrated that compared with control ESCC cells, *ITGAX*-OE ESCC cells had substantially more p-SMAD3 in the membrane and nuclear fractions, accompanied by reduced levels of total SMAD3 in the cytoplasmic fraction (Fig. 4F and Fig. S7E). This result suggests that CD11c promotes the binding of SMAD3 to TGFBR1, facilitating its phosphorylation and subsequent nuclear translocation.

Next, we explored how the CD11c–SMAD3 axis influenced CD80/CD86 expression, as our findings highlighted a previously uncharacterized mechanism regulating CD80/CD86 in epithelial cells. Both *CD80* and *CD86* promoter sequences contain possible p-SMAD3 binding motifs (Fig. S7F and G). Chromatin immunoprecipitation with anti-p-SMAD3 antibody and quantitative PCR assays showed that both *CD80* and *CD86* genes were enriched in ESCC cells, which was significantly enhanced by TGFβ1 (Fig. 4G and Fig. S7H). Furthermore, luciferase reporter assays demonstrated that mutations in the p-SMAD3 binding site of the *CD80* and *CD86* promoters profoundly suppressed their transcriptional activities, suggesting that these regions were critical for basal *CD80* and *CD86* expression in esophageal epithelial cells. Meanwhile, knockdown of *SMAD3* increased their expression by up to 1.5-fold compared to siCtrl (Fig. 4H and Fig. S7G and I). These findings strongly support that p-SMAD3 acted as a transcriptional repressor of *CD80* and *CD86* by interfering with the factors required for activation, thus playing a pivotal role in immune modulation in ESCC.

### CD11c is a potential target and biomarker for ICB therapy

**Fig. 5.**
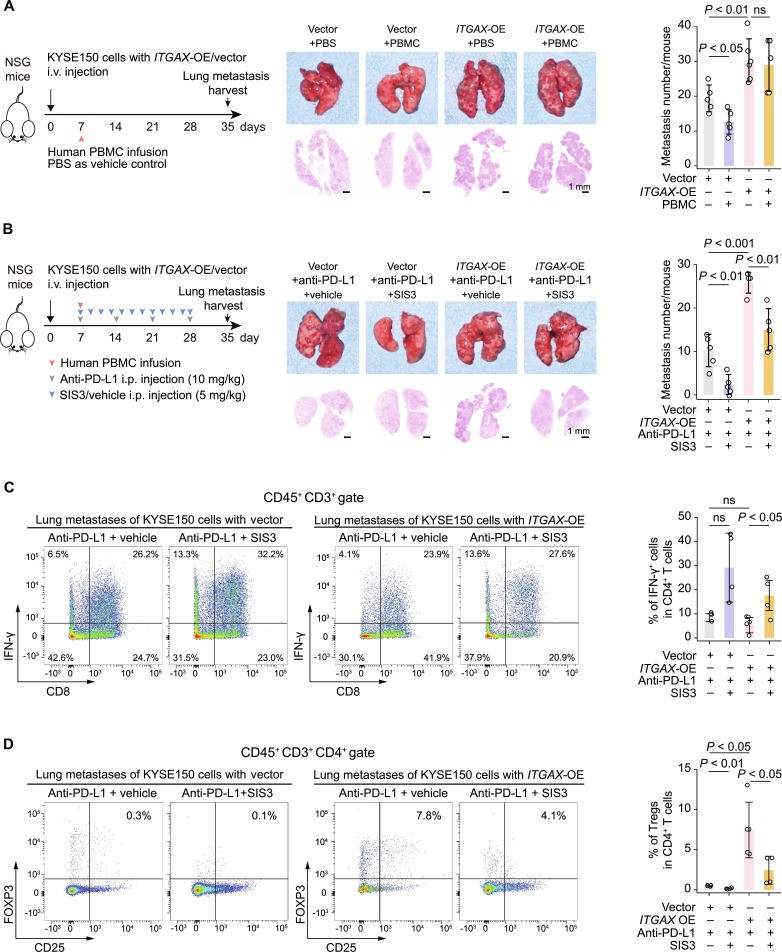
CD11c promotes ESCC cell lung metastasis and immune suppressive TME formation. (A) Effect of *ITGAX*-OE on immune escape in humanized mouse lung metastatic models. Left, schematic of the humanized mouse lung metastasis model. Mice were injected with KYSE150 cells via the tail vein, followed by human PBMCs or PBS (vehicle control) 7 days later. Lung metastases were harvested on day 35. Middle, gross images (top) and H&E staining (bottom) of lung metastasis. Right, quantitative statistics of lung metastasis. The data represent mean ± SD from 5 to 6 mice. (B) Efficacy of anti-PD-L1 antibody treatment in mouse lung metastasis with or without combination treatment with SIS3 under *ITGAX*-OE/vector. Left, schematic of treatment. Middle, gross images (upper) and H&E staining (lower) of lung metastasis. Right, quantitative statistics of lung metastasis. Vehicle (DMSO) treatment groups are the ones with “-“ in SIS3. The data are presented as mean ± SD from 5 mice per group. (C and D) Effect of the treatment of anti-PD-L1 antibody and SIS3 on human IFN-γ^+^CD4^+^ T cells (C) and FOXP3^+^CD25^+^ Tregs (D) in mouse lung metastases. Representative plots (left) and quantitative statistics (right) of flow cytometry. The data represent mean ± SD from 4 to 5 mice. *P* values from Student *t* test. ns, not significant. Abbreviations: Vector, control for OE group; *ITGAX*, integrin alpha X; OE, overexpression; i.v., intravenous injection; i.p., intraperitoneal injection; PBS, phosphate-buffered saline; PBMC, peripheral blood mononuclear cells; PD-L1, programmed death-ligand 1; IFN-γ, interferon-gamma; FOXP3, forkhead box P3.

It is unclear whether CD11c can effectively induce an immunosuppressive TME in vivo. Analysis of scRNA-seq data from patients with ESCC treated with anti-PD-L1 therapy [27] showed that epithelial CD11c expression was higher in unresponsive ESCC patients than in responsive ESCC patients (Fig. S8A). Therefore, we investigated the potential role of CD11c in mediating tumor immune escape and resistance to ICIs in ESCC. We conducted an adoptive cell transfer experiment [35,36] to recapitulate human immune responses in NSG mice. Specifically, human PBMCs were transplanted into mice on day 7 following *ITGAX*-OE or control KYSE150 cell injection, and lung metastases were collected on day 35 (Fig. 5A). This humanized mouse model allowed us to assess the human T cell response in vivo, reducing the confounding effects of myeloid cell-derived AP [37,38]. Results showed no significant changes in body weight between the groups, suggesting the absence of detectable graft-versus-host disease-related effects (Fig. S8B). Histopathology of lung metastatic nodules showed that human PBMC infusion significantly suppressed the growth of control KYSE150 cell-derived metastases, whereas *ITGAX-*OE KYSE150 cells resisted immune attack (Fig. 5A). Moreover, flow cytometry analysis of tumor-infiltrating lymphocytes in lung metastases detected a significant decrease in IFN-γ^+^ CD8 cells (32.86% vs. 13.37%, *P* = 0.0025) and a marginally significant increase in Tregs (FOXP^+^CD25^+^CD4^+^) cells (0.86% vs. 5.11%, *P* = 0.072) in *ITGAX*-OE tumors than in the control tumors (Fig. S8C to E). These findings support the role of CD11c in inducing immunosuppressive T cell responses in tumors, potentially contributing to ICI resistance in ESCCs.

To test whether the immunosuppressive function of CD11c requires p-SMAD3, we implemented anti-phospho-SMAD3 treatment in the aforementioned humanized mouse models. Similarly, the body weight gain of mice was not affected, but the elevated lung metastasis burden was significantly suppressed by SIS3 treatment (Fig. S9A and B). In line with the results shown above, SIS3 treatment significantly reduced Treg cells (2.57% vs. 1.01%, *P* < 0.05) but significantly increased IFN-γ^+^CD4^+^ T cell infiltration (2.25% vs. 8.97%, *P* < 0.05) compared with vehicle control in *ITGAX*-OE metastases (Fig. S9C). We next examined the efficacy of anti-PD-L1 plus SIS3 therapy (Fig. 5B). Notably, *ITGAX*-OE in tumor cells significantly reduced the efficacy of anti-PD-L1 treatment; however, combined SIS3 treatment restored the efficacy of anti-PD-L1 treatment (Fig. 5B) without significantly affecting the body weight gain of mice (Fig. S9D). Moreover, compared with anti-PD-L1 monotherapy, a significant increase in IFN-γ-producing CD4^+^ T cells was observed in *ITGAX*-OE tumors under anti-PD-L1 treatment combined with SIS3 therapy (5.30% vs. 17.63%, *P* < 0.05; Fig. 5C), indicating an elevated immune function. Meanwhile, *ITGAX*-OE tumors exhibited significantly higher Treg cell infiltration than control tumors (7.76% vs. 0.56%, *P* < 0.05). In *ITGAX*-OE tumors, combined SIS3 and anti-PD-L1 treatment significantly reduced the proportion of Treg cells compared with anti-PD-L1 (7.76% vs. 2.56%, *P* < 0.05; Fig. 5D). SIS3 treatment did not alter the abundance or function of the other T cell subsets (Fig. S9E), further suggesting that the combined therapy promoted a more robust anti-tumor immune response in CD11c-expressing tumors, primarily through CD4^+^ T cells. Together, these results demonstrate that ectopic CD11c in epithelial cells confers resistance to ICI therapy in ESCC and that targeting this pathway could enhance the efficacy of ICB. Thus, CD11c may serve as a biomarker and a target for immunotherapy.

### Ectopic CD11c expression in epithelial cells is associated with *TP53* mutations

**Fig. 6.**
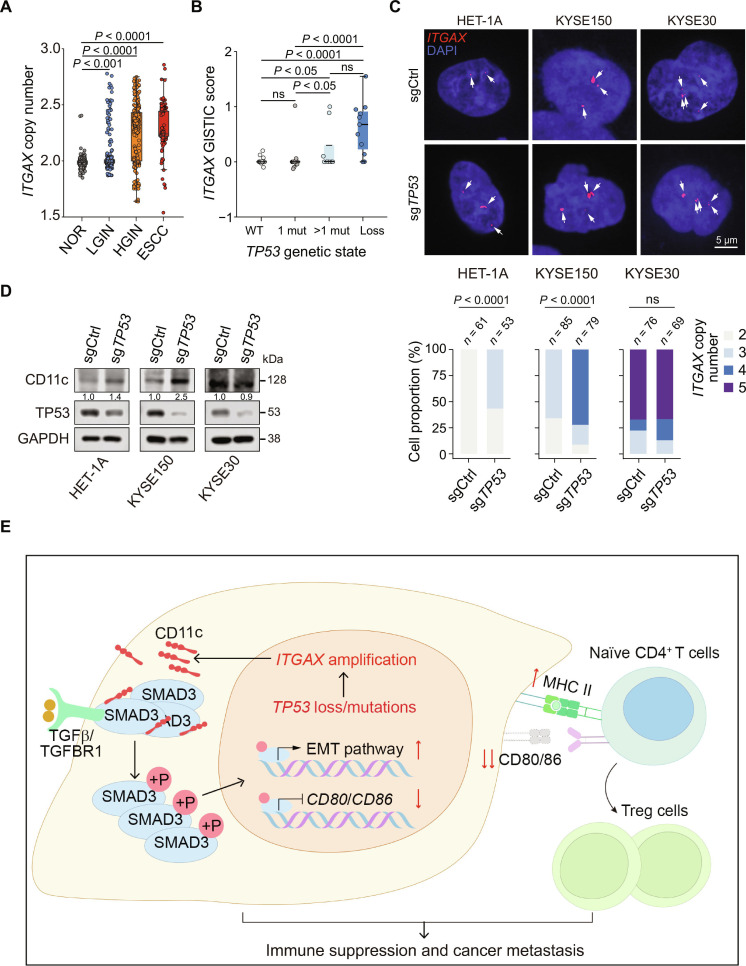
Ectopic CD11c expression in epithelial cells is associated with *TP53* mutations. (A) Copy number variations of the *ITGAX* gene in multistage human ESCC development [25]. Each point indicates a micro-biopsy sample. Sample size at each stage: NOR, 603; LGIN, 245; HGIN, 247; and ESCC, 180. (B) Levels of *ITGAX* copy number (GISTIC score) among human esophageal epithelial clones with different *TP53* states [25]. Each point represents one epithelial clone. Sample size for each group: WT, 35; 1 mut, 24; >1 mut, 8; and Loss, 11. (C) Effect of *TP53-*knockout on *ITGAX* copy number in human esophageal epithelial cells. Top: Representative images of *ITGAX* copy number in *TP53*-unknockout or *TP53*-knockout HET-1A, KYSE150, and KYSE30 cells with *ITGAX*-specific probe (red, arrow pointed). Bottom: Quantitative statistics of the percentage of cells with *ITGAX* copy number in *TP53*-unknockout versus *TP53*-knockout cells. The number of cells (*n*) obtained from 3 independent experiments is indicated on each bar. (D) Immunoblot of CD11c in HET-1A, KYSE150, and KYSE30 cells with or without *TP53* KO. Each experiment had 3 biological repeats. (E) Proposed role of ectopically expressed CD11c for cancer cells to escape immune killing and acquire malignant phenotypes. *TP53* loss-associated *ITGAX* amplification results in CD11c ectopic overexpression in epithelial cells. CD11c interacts with SMAD3 and enhances its binding to TGFβ/TGFBR1, promoting SMAD3 phosphorylation. Hyperactivated SMAD3 subsequently translocates to the nucleus, where it suppresses *CD80/CD86* transcription while up-regulating the levels of MHC class II molecules. Together, these changes impair tumor cell-mediated antigen presentation and induce EMT, thereby promoting cancer development. In (A) to (C), *P* values were from the Wilcoxon rank-sum test. ns, not significant. Abbreviations: *ITGAX*, integrin alpha X; NOR, normal epithelial tissue; INF, inflammatory tissue; LGIN, low-grade intraepithelial neoplasia tissue; HGIN, high-grade intraepithelial neoplasia tissue; ESCC, esophageal squamous cell carcinoma; GISTIC, Genomic Identification of Significant Targets in Cancer; WT, wild type; 1 mut, one mutation; >1 mut, multiple mutations; Loss, *TP53* biallelic loss; Ctrl, control; EMT, epithelial–mesenchymal transition; SMAD3, mothers against decapentaplegic homolog 3; P, phosphorylation; TGFβ, transforming growth factor beta; TGFBR1, transforming growth factor beta receptor I; Treg, regulatory T cell; MHC II, major histocompatibility complex class II.

Finally, we sought to understand the mechanisms underlying the aberrant CD11c expression in esophageal epithelial cells. Because our previous genomic analysis showed recurrent amplification of chromosome 16p11.2 (harboring *ITGAX*) specifically in TP53 biallelic loss clones [25], we hypothesized that CD11c overexpression may arise from genomic alterations. Genomic alteration analysis of *the ITGAX* locus in human multistage esophageal precancerous and cancerous samples [25] showed no somatic mutations; however, the copy number of *ITGAX* significantly increased during ESCC progression (Fig. 6A). *TP53* inactivation mutations are responsible for many ESCC copy number alterations [25]; therefore, we investigated whether *ITGAX* amplification was correlated with *TP53* mutations. The results demonstrated that clones with *TP53* biallelic loss or multiple mutations had significantly higher *ITGAX* copy numbers than those with the wild type or only one mutation in *TP53* (Fig. 6B). Furthermore, *TP53*-knockout in immortalized esophageal cells (HET-1A) and ESCC cells (KYSE150 and KYSE30) confirmed that *TP53-*knockout increased *ITGAX* copy numbers (Fig. 6C). *ITGAX* copy numbers were significantly increased in more than half of the *TP53*-knockout HET-1A cells (3 copies in 56.60% of cells) compared to those of *TP53*-unknockout control HET-1A cells (2 copies in 100% of cells; *P* < 0.001). Similarly, KYSE150 cells (known with *TP53* R248Q mutation) showed significantly increased *ITGAX* copies when *TP53* was knocked out (72.15% had 4 copies, 18.99% had 3 copies, and 8.86% had 2 copies) compared to those of their control counterparts (65.88% had 3 copies, 34.12% had 2 copies, *P* < 0.001). *TP53*-knockout did not significantly alter the *ITGAX* copy number in KYSE30 cells, a cell line that already harbors multiple *TP53* mutations (*TP53*-unknockout: 67.11% had 5 copies, 10.53% had 4 copies, and 22.37% had 3 copies; *TP53*-knockout: 66.67% had 5 copies, 20.29% had 4 copies, and 13.04% had 3 copies, *P* = 0.74; Fig. 6C). Additionally, *TP53-*knockout in HET-1A and KYSE150 cells substantially elevated CD11c protein levels; however, in KYSE30 cells with high levels of CD11c, *TP53-*knockout did not significantly alter the CD11c protein level (Fig. 6D), in line with the *ITGAX* copy number alteration. These results support the hypothesis that *TP53* inactivation drives *ITGAX* gene amplification, thereby causing CD11c overexpression in malignant esophageal epithelial cells, contributing to immune evasion and metastasis.

## Discussion

Escaping immune surveillance is critical in cancer initiation and progression. Although numerous mechanisms have been proposed, the mechanism by which cancer cells escape immune killing is unclear [39]. In this study, multiomics analysis revealed that CD11c, a protein usually expressed by myeloid cells, was also expressed in transformed esophageal epithelial cells during ESCC tumorigenesis. CD11c expression in immune cells is involved in immune responses, such as fibrinogen receptor-mediated cell–cell interactions and chemokine responses during inflammation [40,41]; however, the role of CD11c in ESCCs remains unclear. Notably, we demonstrated that in epithelial cancer cells, ectopically expressed CD11c promoted SMAD3 phosphorylation and activated TGFβ signaling, suppressing costimulatory factor expression and inducing EMT, resulting in immune escape and malignant phenotypes of cancer cells (Fig. 6E). Our findings reveal a previously unrecognized role of CD11c in driving immune evasion and malignancy in ESCC, highlighting the role of the CD11c–SMAD3 axis in ESCC tumorigenesis. Furthermore, we found that CD11c-OE in epithelial cells is likely caused by *TP53* inactivation, which is a critical and early event in ESCC and other cancers [25,42]. This distinct AP underscored the necessity of exploring the CD11c–SMAD3 axis as a potential biomarker and therapeutic target in ESCC, especially for patients with *TP53* mutations who may exhibit resistance to current immune therapies.

CD11c expression and functional role in epithelial cells remain poorly characterized. In this study, we observed that CD11c is ectopically expressed in esophageal epithelial cells, with its prevalence increasing from precancerous lesions to invasive ESCC, prompting us to investigate its potential role in ESCC progression. We have previously reported that chromosome 16p11.2, where the *ITGAX* gene is located, is often amplified in precancerous and cancerous *TP53*-inactivated esophageal epithelium [25]. Here, we confirmed that *TP53-*knockout induces amplification of the *ITGAX* locus and subsequent CD11c overexpression in both immortalized esophageal cells and ESCC cell lines. *TP53* mutations are among the most prevalent somatic mutations in various cancers, including ESCC, which drive genomic instability, alter cell fate, and impair the immune response [25,43,44]. Hence, the abnormal AP process caused by ectopic CD11c epithelial expression suggests that *TP53* mutations may influence the TME in previously unrecognized ways, pointing to possible additional roles of *TP53* in cancer. However, whether *ITGAX* amplification is dependent on tissue or cell type, how *TP53* inactivation may select *ITGAX* for amplification, and whether there are other mechanisms leading to the regulation of *TP53* on CD11c all remain open questions that require further investigation.

Several studies have indicated that enhancing AP can increase cancer cell sensitivity to immunotherapy [45,46]. For instance, CD80/CD86 is essential for the T cell immune response, and agents that up-regulate CD80 and CD86 restore the immune response to tumor cells [47,48]. However, the molecular mechanisms underlying low CD80/CD86 levels in cancer cells remain unclear. Here, we demonstrated that ectopic CD11c expression in epithelial cancer cells contributes to an immunosuppressive state characterized by high MHC class II but low CD80/CD86 expression [45] through p-SMAD3 activation. Moreover, high CD11c levels in ESCC cells were associated with resistance to anti-PD-L1 treatment. Although immunotherapies largely rely on ICIs to enhance effective T cell activity, the benefits of PD-L1 as a predictive biomarker for ICI therapy are limited in ESCC because of its low PD-L1 levels [22,49]. Previous studies have linked immune checkpoint therapy to enhanced TGFβ-SMAD3 signaling or EMT-like therapy-resistant tumor states [12,13]. However, these findings largely reflect immune-contextual or phenotypic associations in which tumor cells are viewed as downstream responders to immune modulation rather than as active integrators of EMT and antigen-presentation programs. In this study, we identified a CD11c–SMAD3 axis that directly couples EMT activation with suppression of AP within epithelial tumor cells. Given the central role of TGFβ signaling in EMT, our findings suggested that targeting TGFβ activity or its downstream signaling can increase immune surveillance and inhibit the invasiveness and metastatic potential associated with EMT, thus improving tumor cell clearance [50,51]. Consistent with a previous report on SIS3 for cancer treatment in mice [52], anti-PD-L1 plus SIS3 treatment can effectively suppress lung metastasis of CD11c^+^ cancer. Therefore, targeting the CD11c–SMAD3 axis may provide a new avenue for ESCC immunotherapy by reshaping the metastatic microenvironment. A deeper understanding of the molecular basis of this interaction may ultimately facilitate the rational design of therapeutic interventions targeting dysregulated TGFβ/SMAD3 signaling.

In summary, the present study revealed that during ESCC initiation and progression, a proportion of esophageal epithelial cells aberrantly express CD11c, a molecule usually expressed in myeloid cells, likely due to *TP53* mutations in cancer cells. Ectopic CD11c expression in transformed epithelial cells facilitates cancer cells in escaping immune killing and acquiring malignant phenotypes by activating SMAD3 signaling. Further anti-SMAD3 treatment effectively enhances CD11c-induced immunotherapy resistance. These findings provide a new insight into complicated immune reprogramming in cancer cells and provide a possible clue for developing anticancer immunotherapies.

## Conclusions

This study uncovered a mechanism by which a cluster of *TP53*-loss ESCC cells exploits ectopic CD11c expression to promote immune evasion and drive malignant phenotypes through CD11c-mediated SMAD3 phosphorylation, which, in turn, suppresses costimulatory molecules CD80/CD86 and activates EMT. These findings highlight the potential of combining anti-SMAD3 treatment with immunotherapy as a therapeutic strategy for ESCC.

## Ethical Approval

Surgically resected human ESCC and their adjacent normal tissues were collected from patients at Cancer Hospital, Chinese Academy of Medical Sciences (*n* = 11), with approval from the Institutional Review Boards of Cancer Hospital, Chinese Academy of Medical Sciences (NCC2022C141). All patients provided written informed consent. Animal experiments were carried out in conformity with approved ethical protocols and guidelines from the Institutional Animal Care and Use Committee of the Chinese Academy of Medical Sciences (NCC2021A271).

## Data Availability

Origins of public omics’ datasets are noted when used in the main text and are listed as follows: Multistage esophageal tissue samples’ WES and WGS data [25] are deposited in GSA-Human (Genome Sequence Archive in BIG Data Center, Beijing Institute of Genomics, Chinese Academy of Sciences, https://bigd.big.ac.cn/gsa-human) as PRJCA015964. Multistage esophageal tissue samples’ single-cell RNA-seq and spatial RNA-seq data [14] can be accessed under HRA000776 in GSA-Human. Multistage esophageal tissue samples’ proteome and phosphoproteome data [26] can be reached through PXD038961 in the ProteomeXchange Consortium. ESCC bulk RNA-seq data [16] are available in GEO with accession number GSE160269. The scRNA-seq data of pretreatment ESCC patients [27] are in GSA-Human under accession number HRA003591. TCGA data, which support the findings of this study, are available from the TCGA database (http://cancergenome.nih.gov) and TumorPortal (http://www.tumorportal.org) with the project ID “TCGA-ESCA”. All other remaining data are available within the article and Supplementary Materials. Any additional information or code required to re-analyze the data reported in this paper is available upon request.

## Supplementary Materials

Supplementary 1` Figs. S1 to S9 Tables S1 to S5
