# Supplementary material for: Ectopic CD11c Drives SMAD3-Mediated Aberrant Antigen Presentation and Epithelial–Mesenchymal Transition in Esophageal Squamous Cell Carcinoma
Source: Cancer Commun (Lond). 2026 Mar 6;46:0014. doi: 10.34133/cancomm.0014 (PMC12963642; doi:10.34133/cancomm.0014)
Supplement: Supplementary 1` — Figs. S1 to S9 Tables S1 to S5 [file cancomm.0014.f1.pdf]

Supplementary Materials for

Ectopic CD11c drives SMAD3-mediated aberrant antigen presentation and epithelial-mesenchymal transition in esophageal squamous cell carcinoma

Han Liao[1,†], Xuan Zhao[1,†], Liping Chen[1], Qingyi Liu[1], Chenying Li[1], Kai Li[1], Yiyi Xi[1], Yanrong Shen[1], Wen Tan[1,2], Chen Wu[1,2,3,4,*], and Dongxin Lin[1,2,3,5,*]

Affiliations

[1]Department of Etiology and Carcinogenesis, National Cancer Center/National Clinical Research Center/Cancer Hospital, Chinese Academy of Medical Sciences (CAMS) and Peking Union Medical College (PUMC), Beijing 100021, P. R. China.

[2]Key Laboratory of Cancer Genomic Biology, Chinese Academy of Medical Sciences and Peking Union Medical College, Beijing 100021, P. R. China.

[3]Collaborative Innovation Center for Cancer Personalized Medicine, Nanjing Medical University, Nanjing 211166, Jiangsu, P. R. China.

[4]Chinese Academy of Medical Sciences Oxford Institute, Chinese Academy of Medical Sciences, Beijing 100006, P. R. China.

[5]State Key Laboratory of Oncology in South China, Sun Yat-sen University Cancer Center, Guangzhou 510060, Guangdong, P. R. China.

[†]Han Liao and Xuan Zhao contributed equally to this study.

*Correspondence

Dongxin Lin (lindx@cicams.ac.cn)

Department of Etiology and Carcinogenesis, National Cancer Center/National Clinical Research Center/Cancer Hospital, Chinese Academy of Medical Sciences (CAMS) and Peking Union Medical College (PUMC), Beijing 100021, P. R. China.

Chen Wu (chenwu@cicams.ac.cn)
Department of Etiology and Carcinogenesis, National Cancer Center/National Clinical Research Center/Cancer Hospital, Chinese Academy of Medical Sciences (CAMS) and Peking Union Medical College (PUMC), Beijing 100021, P. R. China.

The file includes:

Supplementary Figures S1 to S9

Supplementary Tables S1 to S5

Liao et al. Supplementary Figure S1

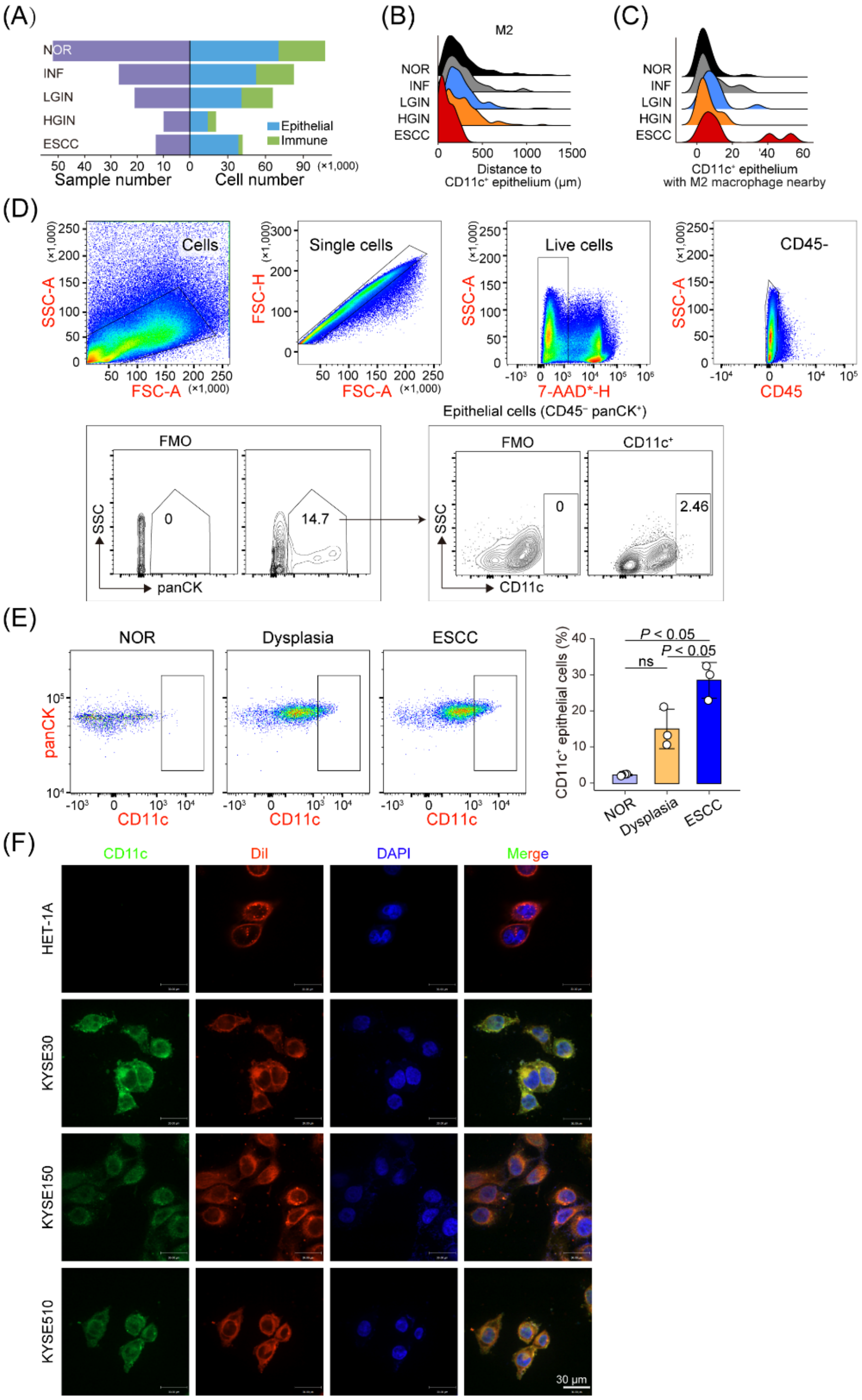

Supplementary Figure S1. Further identification and spatial distribution of CD11c⁺ epithelial cells across different histopathological stages in ESCC development. (A) Sample number of tissue sections at each stage included in multiplexed immunofluorescence assays

(left) and epithelial and immune cell numbers that can be effectively distinguished after fluorescence signal processing in samples of the corresponding stage (right). **(B)** The distribution of spatial distance between M2 macrophages and $CD11c^+$ epithelial cells across different histopathological stages of ESCC development, using density, with the x-axis indicating the distance to the nearest $CD11c^+$ epithelial cell from the M2 macrophage. **(C)** The number distribution of $CD11c^+$ epithelial cells having at least one M2 macrophage within a radius of 100 μm. **(D)** The flow cytometry gating and analyzing strategy of the mouse esophageal precancerous or ESCC lesion. **(E)** Flow cytometry of mouse esophageal precancerous or ESCC lesion (left) and quantitative statistics of $CD11c^+$ cells per epithelial cells (right). The data present mean ± SD from 3 to 4 mice. *P* values were from Student's *t*-test. ns, not significant. **(F)** The immunofluorescence staining and confocal microscopy imaging of CD11c in human normal esophageal cells HET-1A and ESCC cells KYSE30, KYSE150 and KYSE510. Color channels, CD11c (green); DiI (red, for cell membrane); DAPI (blue, for cell nuclei). Abbreviations: NOR, normal epithelial tissue; INF, inflammatory tissue; LGIN, low-grade intraepithelial neoplasia tissue; HGIN, high-grade intraepithelial neoplasia tissue; ESCC, esophageal squamous cell carcinoma; panCK, pan-cytokeratin; SD, standard deviation.

Liao et al. Supplementary Figure S2

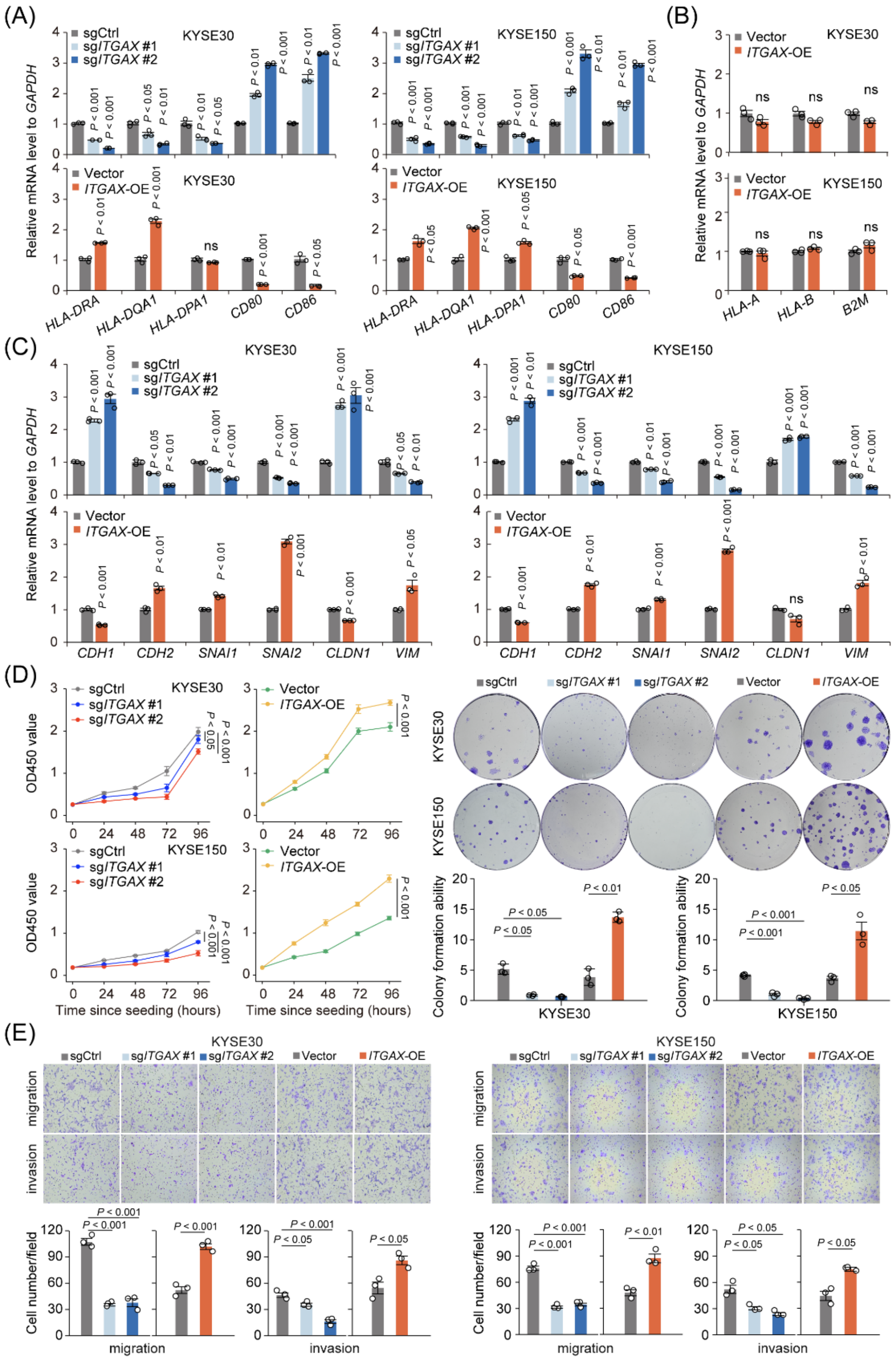

Supplementary Figure S2. CD11c expression in esophageal epithelial cells promotes ESCC formation and progression. (A) The effects of *ITGAX* KO and OE on the mRNA expression of the key AP (MHC class II and co-stimulatory molecules) in KYSE30 and KYSE150 cells. **(B)**The

effects of *ITGAX* OE on the mRNA expression of the MHC class I in KYSE30 and KYSE150 cells. **(C)** The effects of *ITGAX* KO and OE on the mRNA expression of the EMT pathway genes in KYSE30 and KYSE150 cells. **(D)** The effects of *ITGAX* KO or OE on KYSE30 and KYSE150 proliferation (left) and colony formation (right). **(E)** The effects of *ITGAX* KO or OE on KYSE30 and KYSE150 cell migration and invasion in vitro. The data present mean ± SEM from 3 experiments, and each had 3 biological repeats. *, $P < 0.05$; **, $P < 0.01$; ***, $P < 0.001$; ns, not significant from Student's *t*-test. *P* values shown above each group were calculated by comparing that group with the sgCtrl or Vector group. Abbreviations: *ITGAX*, integrin alpha X; sgCtrl, single guide RNA control; sg*ITGAX*, single guide RNA targeting *ITGAX* gene; Vector, control for OE group; OE, overexpression; KO, knockout.

Liao et al. Supplementary Figure S3

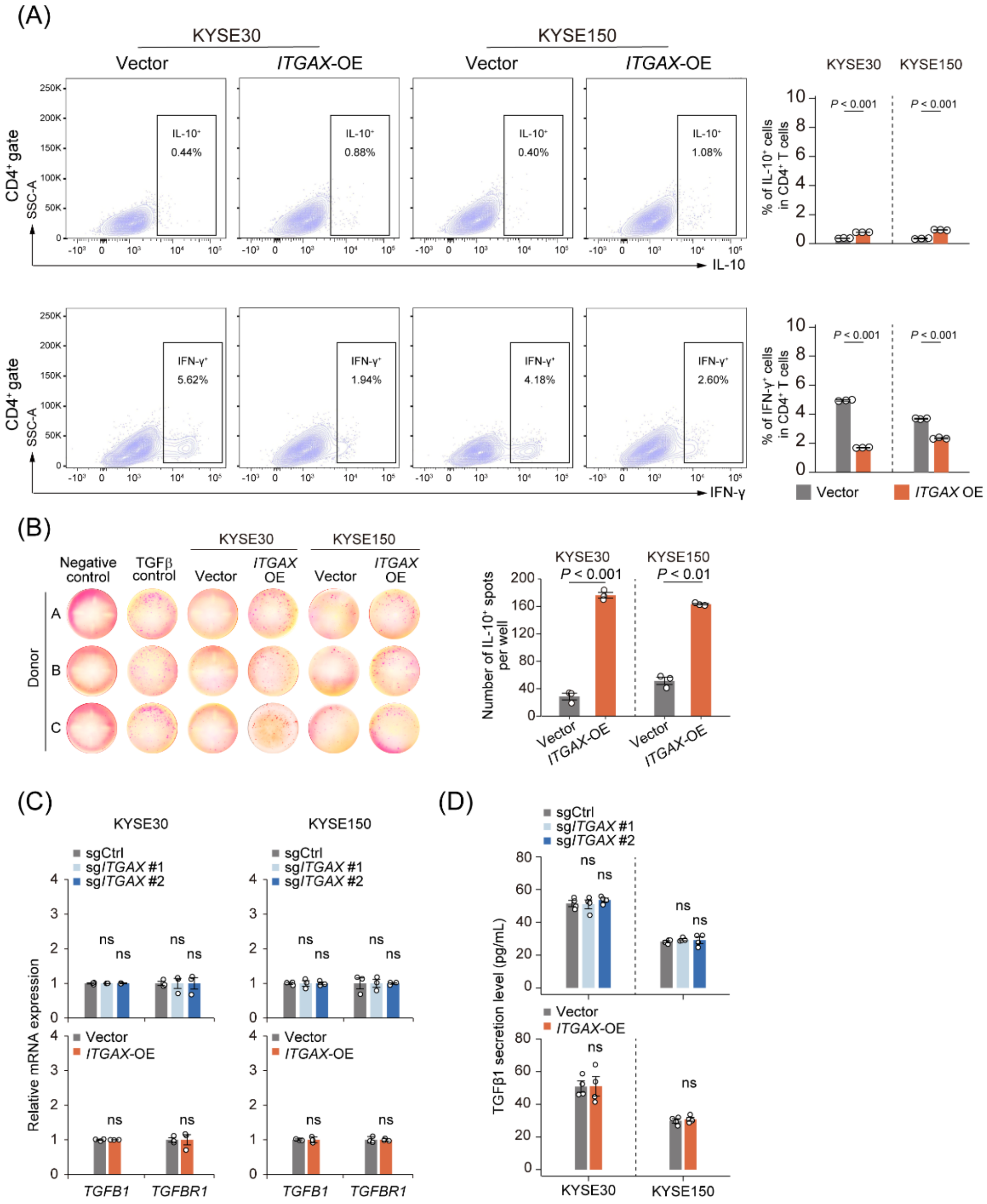

Supplementary Figure S3. Effect of CD11c in immune evasion in vitro. **(A)** Effect of *ITGAX*-OE in KYSE30 and KYSE150 on IL-10 and IFN-γ expression in $CD4^+$ T cells. Left, flow cytometry examples. Right, quantification of IL-10^+ and IFN-γ^+ CD4 T cells in cocultured human naïve

CD4$^+$ T cells determined by flow cytometry analysis. **(B)** Effect of *ITGAX*-OE in KYSE30 and KYSE150 cells on IL-10 expression in cocultured human naïve CD4$^+$ T cells determined by ELISpot assay. Negative control, unstimulated control; TGFβ1 control, positive control. Left, ELISpot images showing the IL-10 positive spots in red. Right, quantitative statistics of IL-10$^+$ spot. **(C)** Effect of *ITGAX* KO and OE on *TGFB1* and *TGFBR1* expression. **(D)** ELISA results on TGFβ1 secretion level under conditions of *ITGAX* KO and OE in KYSE30 and KYSE150 cell lines. The data present mean ± SEM from 3 experiments, and each had 3 biological repeats. ns, not significant for Student's *t*-test. *P* values shown above each group were calculated by comparing that group with the sgCtrl or Vector group. Abbreviations: *ITGAX*, integrin alpha X; sgCtrl, single guide RNA control; sg*ITGAX*, single guide RNA targeting *ITGAX* gene; Vector, control for OE group; OE, overexpression; TGFβ, transforming growth factor beta; TGFB1, transforming growth factor beta 1; TGFBR1, transforming growth factor beta receptor I; IL-10, interleukin-10; IFN-γ, interferon γ; KO, knockout.

Liao et al. Supplementary Figure S4

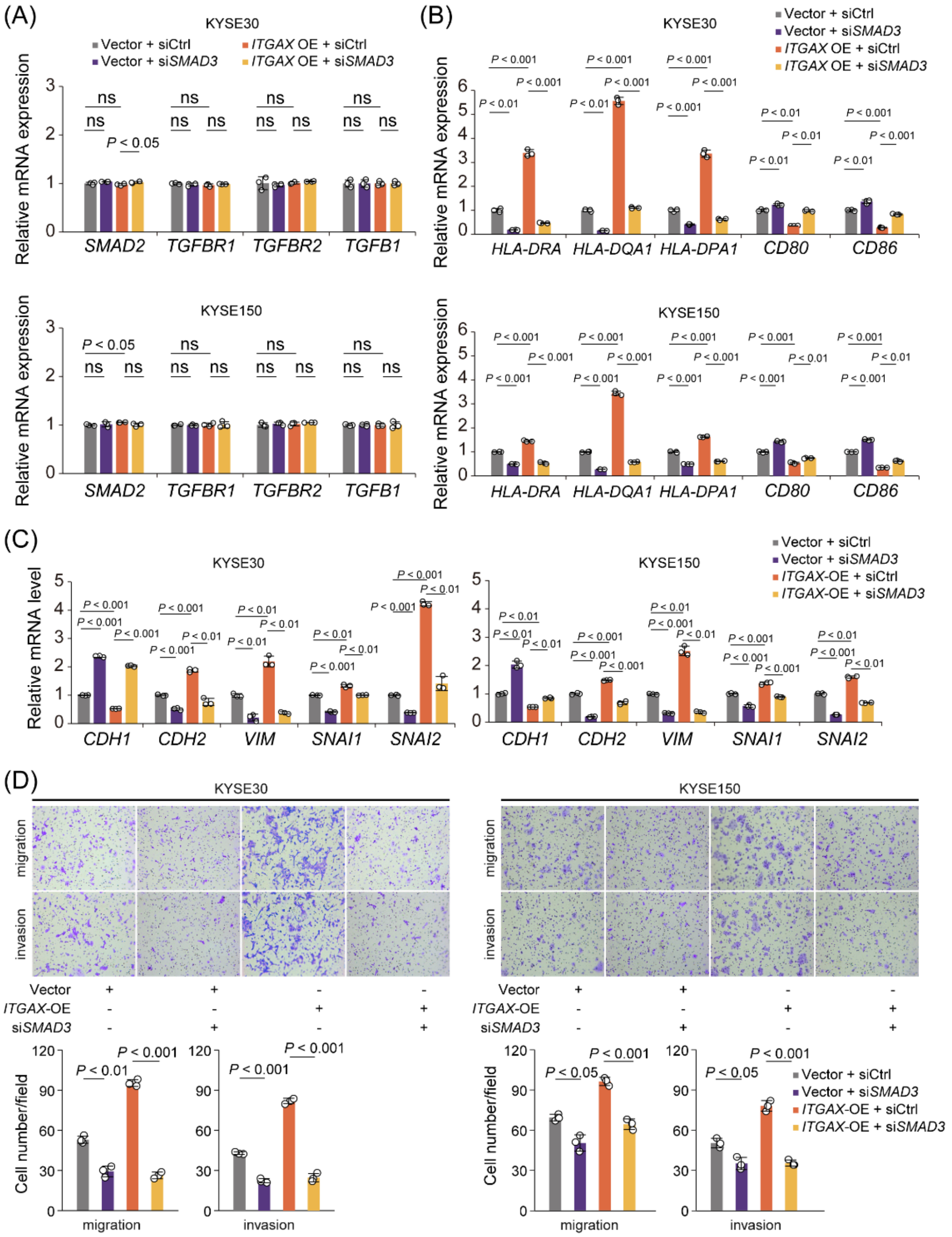

Supplementary Figure S4. CD11c expression in epithelial cells induces a suppressive antigen-presenting process and epithelial-mesenchymal transition via SMAD3. (A) The effects of *SMAD3* knockdown on the expression of the TGFβ pathway key genes *SMAD2*,

TGFBR1, *TGFBR2*, *and TGFB1* in KYSE30 and KYSE150 cells with or without *ITGAX* OE. **(B)** The effects of *SMAD3* knockdown on the MHC class II genes (*HLA-DRA*, *HLA-DQA1*, *HLA-DPA1*) and the co-stimulatory genes (*CD80*, *CD86*) in KYSE30 and KYSE150 cells with or without *ITGAX* OE. **(C)** The effects of *SMAD3* knockdown on the key genes in the EMT pathway (*CDH1*, *CDH2*, *VIM*, *SNAI1*, *SNAI2*) in KYSE30 and KYSE150 cells with or without *ITGAX* OE. **(D)** The effects of *SMAD3* knockdown on the migration and invasion ability of KYSE30 and KYSE150 cells with or without *ITGAX* OE. The lower panels show representative Transwell images. Data represent mean ± SEM from 3 independent experiments and each had 3 biological repeats. *, $P < 0.05$; **, $P < 0.01$; ***, $P < 0.001$ from Student's *t*-test. Abbreviations: *ITGAX*, integrin alpha X; sgCtrl, single guide RNA control; sg*ITGAX*, single guide RNA targeting *ITGAX* gene; Vector, control for OE group; OE, overexpression; siCtrl, siRNA control; si*SMAD3*, siRNA knockdown for *SMAD3*.

Liao et al. Supplementary Figure S5

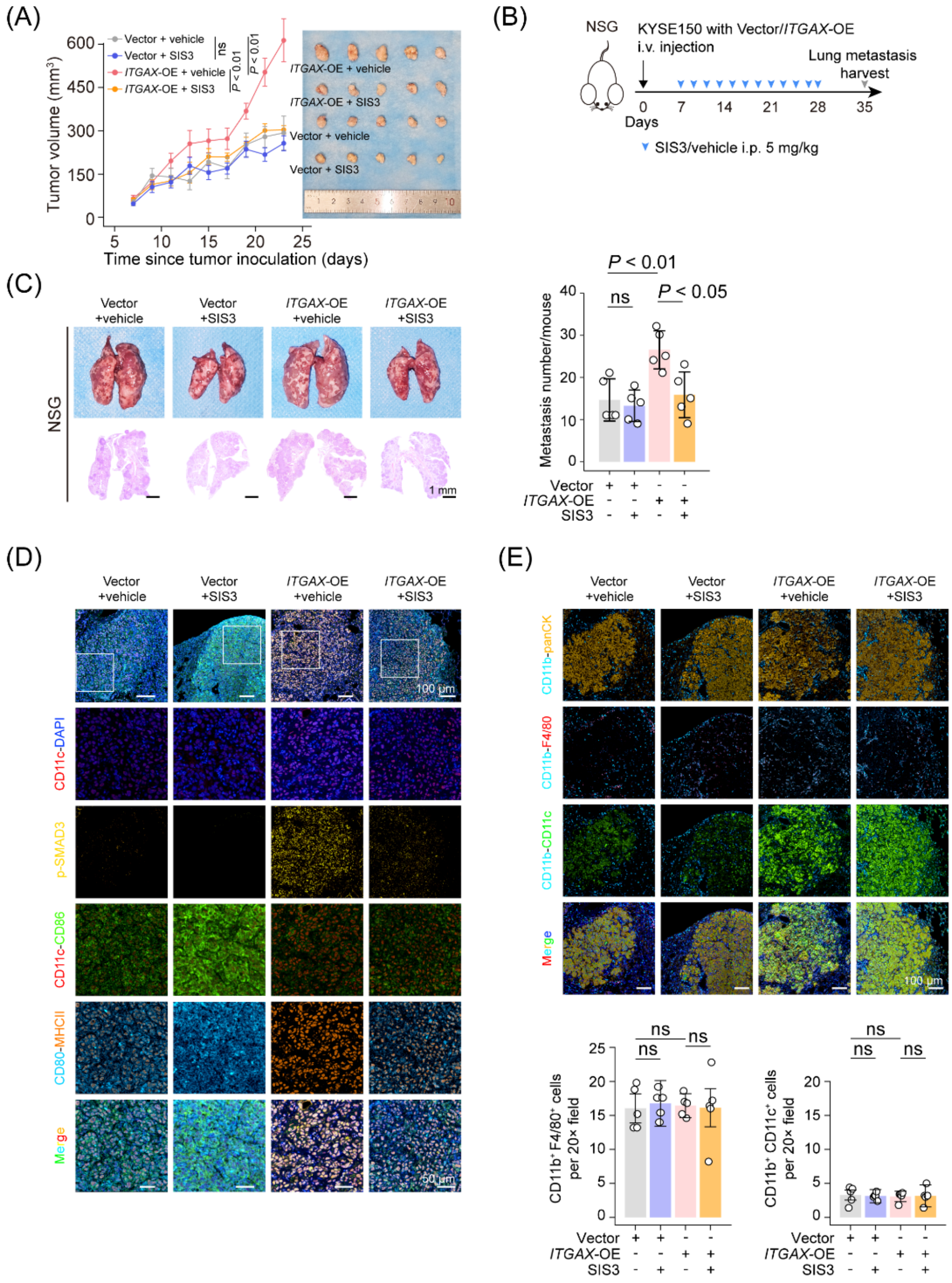

Supplementary Figure S5. Anti-p-SMAD3 in vivo treatment under different *ITGAX* conditions. (A) Effects of *ITGAX* OE and p-SMAD3 level on mouse subcutaneous xenografts growth of KYSE150 cells. Left, tumor size at different time points. Right, representative

image of tumor. **(B)** Schematic of lung metastasis experimental design with *ITGAX* OE and SIS3 treatments. **(C)** Results from the experiments in (B). Left, gross images (upper) and H&E staining (lower) of lung metastasis. Right, quantitative statistics of lung metastasis number. Data represent mean ± SD from 5 mice. **(D)** mIF images of lung metastasis from samples in (B) and (C) stained with DAPI (blue), CD11c (red), p-SMAD3 (yellow), CD86 (green), *CD80* (aqua blue) and MHCII (orange). All rows from the second row are the zoomed-in region of the white rectangles in the first row. Channels are illustrated as colored texts. **(E)** Upper panel, mIF images of lung metastasis from samples in (B) and (C) stained with panCK (yellow) and CD11b (aqua blue), F4/80 (red) and CD11c (green). Lower panel, quantification for counts of canonical macrophages (CD11b^{+}F4/80^{+}) and dendritic cells (CD11b^{+}CD11c^{+}) in per 20× microscopic field. Data represent mean ± SD. ns, not significant from Student's *t*-test. Abbreviations: *ITGAX*, integrin alpha X; Vector, control for OE group; OE, overexpression; i.v., intravenous injection; i.p., intraperitoneal injection; panCK, pan-cytokeratin; p-SMAD3, phosphorylated SMAD3; mIF, multiplex immunofluorescence; MHCII, MHC-II protein antibodies.

Liao et al. Supplementary Figure S6

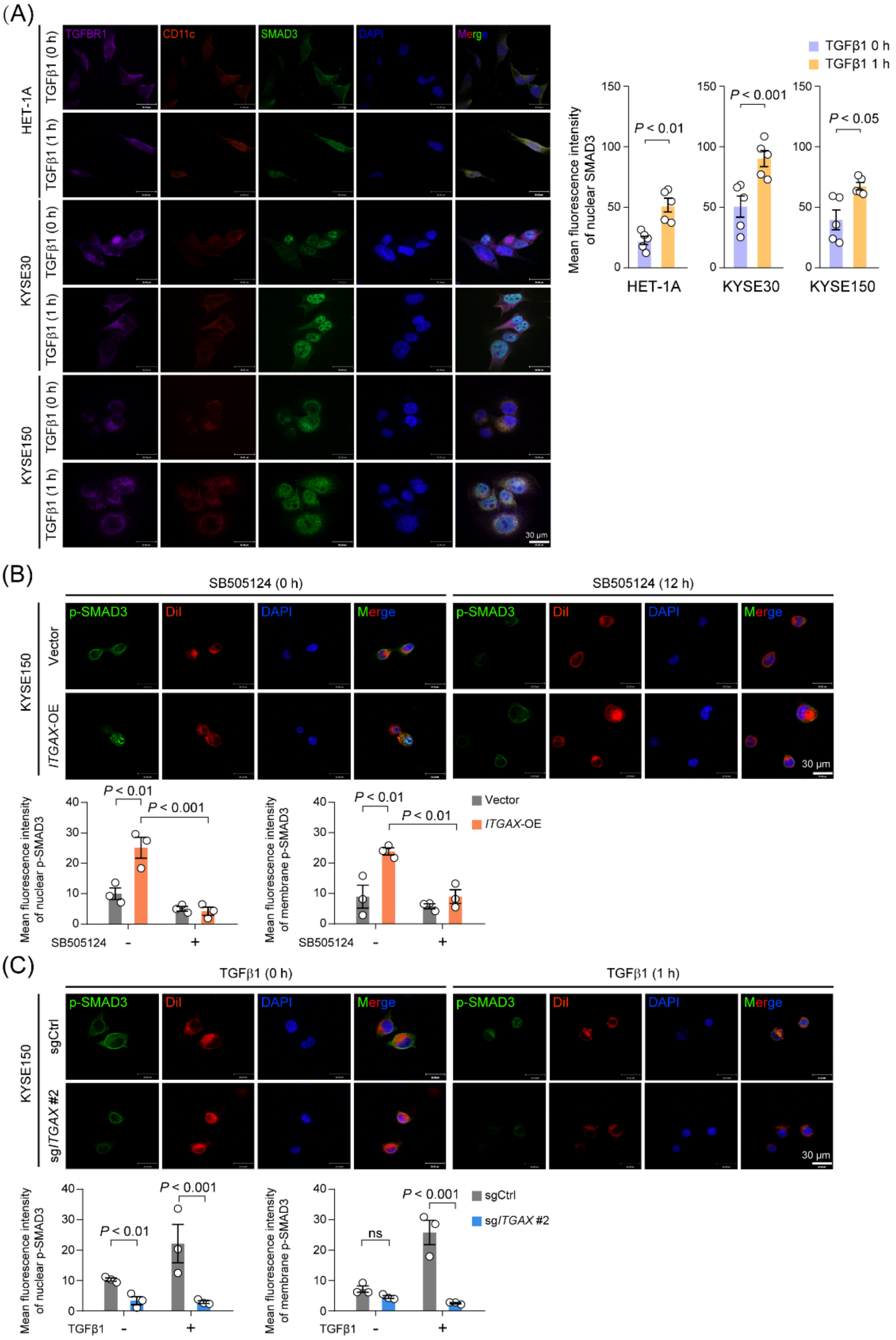

Supplementary Figure S6. CD11c facilitates SMAD3 nuclear translocation via direct binding to phosphorylated SMAD3. (A) Left, representative immunofluorescence images showing

co-localization of SMAD3, TGFBR1 and CD11c in normal human esophageal epithelial cells (HET-1A) and ESCC cells (KYSE30 and KYSE150) in vitro in the presence (1 h) or absence (0 h) of TGFβ1. Right, quantitative results of mean fluorescence intensity of nuclear SMAD3 with or without TGFβ1 treatment. **(B)** The effect of *ITGAX* OE on SMAD3 phosphorylation in KYSE150 cells in the absence (0 h) or presence (12 h) of TGFBR1 inhibitor SB505124. Upper panel, representative immunofluorescence images. Lower panel, quantitative results of mean fluorescence intensity of p-SMAD3. **(C)** The effect of *ITGAX* KO on SMAD3 phosphorylation in KYSE150 cells in the absence (0 h) or presence (1 h) of TGFβ1. Upper panel, representative immunofluorescence images. Lower panel, quantitative results of mean fluorescence intensity of p-SMAD3. Data represent mean ± SEM from 3 independent experiments. *, $P < 0.05$; **, $P < 0.01$; ***, $P < 0.001$; ns, not significant from Student's *t*-test. Abbreviations: *ITGAX*, integrin alpha X; sgCtrl, single guide RNA control; sg*ITGAX*, single guide RNA targeting *ITGAX* gene; Vector, control for OE group; OE, overexpression.

Liao et al. Supplementary Figure S7

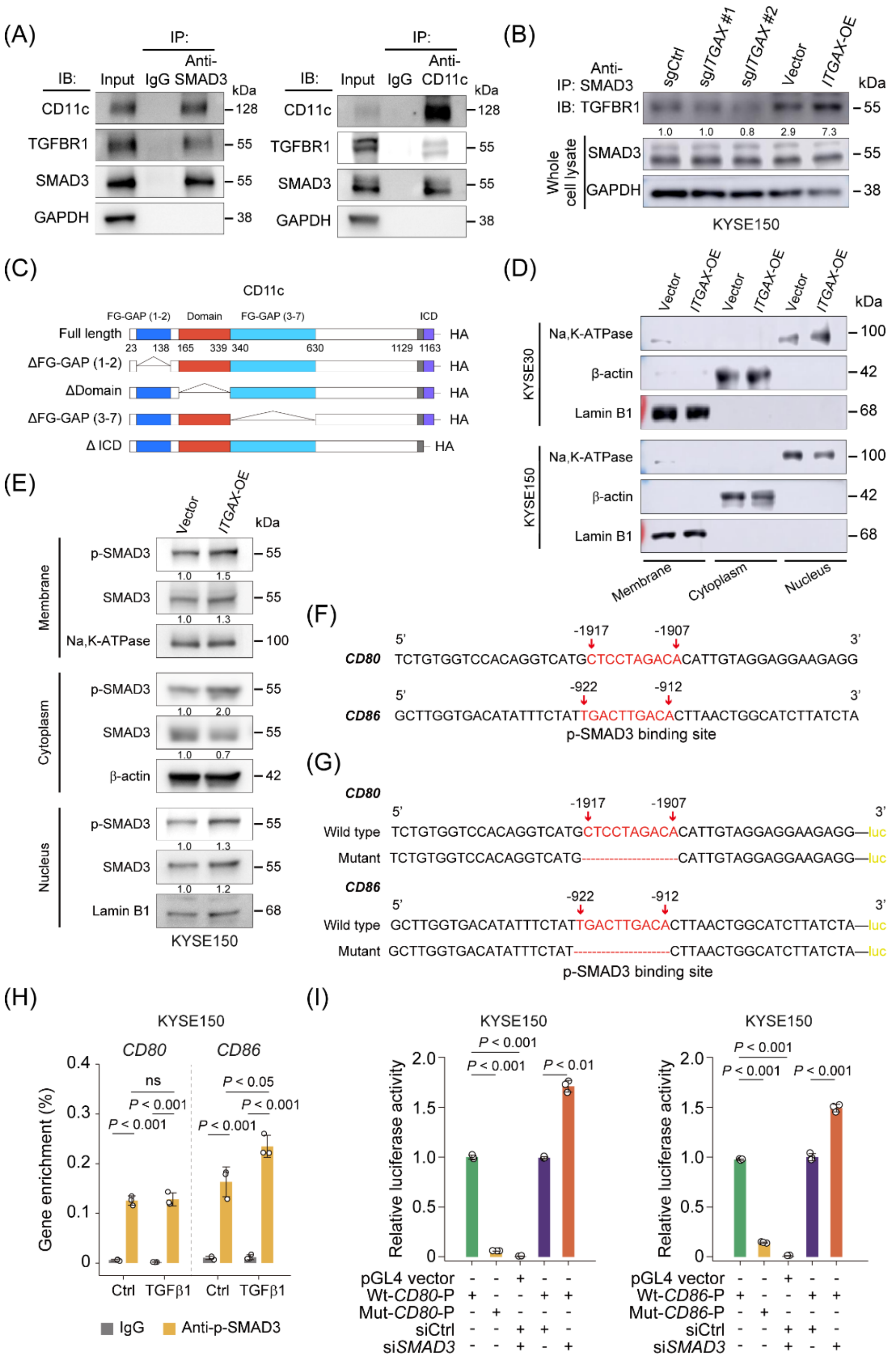

Supplementary Figure S7. SMAD3 suppresses CD80/CD86 expression by directly inhibiting the activity of the *CD80*/*CD86* gene promoters. (A) Western blot analysis of the co-

immunoprecipitation products collected from whole cell lysate with SMAD3 antibody (left) or CD11c antibody (right) in KYSE150 cells. **(B)** Western blot analysis of immunoprecipitation products collected using SMAD3 antibody shows the interaction of TGFBR1-SMAD3 in KYSE150 cells with *ITGAX* KO or OE. **(C)** Design of CD11c truncation mutants. Truncated constructs were designed based on the UniProt (https://www.uniprot.org/)-annotated domains of human *ITGAX*, including the extracellular repeat regions (FG-GAP 1–7, blue and cyan), the von Willebrand factor A-like domain (vWFA domain, red), and intracellular topological domain (purple), with truncations generated along these domain boundaries. **(D)** Western blotting analysis shows the efficiency of different subcellular component separation. **(E)** Cell membrane (marked by Na, K-ATPase), cytoplasm (marked by β-actin) and nucleus (marked by Lamin B1) fractions of *ITGAX*-OE KYSE150 cells were collected and subjected to western blotting analysis of p-SMAD3 and SMAD3. **(F)** The promoter sequences of human *CD80* (upper panel) and *CD86* (lower panel) show a potential p-SMAD3 binding site (indicated in red). **(G)** DNA sequences of truncated or deleted SMAD3 binding motifs in the *CD80* and *CD86* promoter regions for constructing the pGL4 reporter plasmids. **(H)** ChIP-qPCR assays show enrichment of *CD80* or *CD86* promoter sequences in chromatin immunoprecipitated with anti-p-SMAD3 antibody from KYSE150 cells treated with vehicle (Ctrl) or TGFβ1. **(I)** Luciferase reporter assays in KYSE150 cells using indicated reporter plasmid of *CD80* (left panel) or *CD86* (right panel) promoter or siRNA targeting *SMAD3*. Data are mean ± SEM from 3 experiments and each had 3 replicates. *P*-values were from Student's *t*-test. Abbreviations: *ITGAX*, integrin alpha X; sgCtrl, single guide RNA control; sg*ITGAX*, single guide RNA targeting *ITGAX* gene; Vector, control for OE group; OE, overexpression; Δ, deletion; FG-GAP (1-2), Phenylalanine-Glycine-GAP repeat fragment 1 to 2; FG-GAP (3-7), Phenylalanine-Glycine-GAP repeat fragment 3 to 7; ICD, intracellular

domain; Wt-*CD80*-P, wild-type *CD80* promoter; Mut-*CD80*-P, mutant-type *CD80* promoter (without p-SMAD3 binding motif); Wt-*CD86*-P, wild-type *CD86* promoter; Mut-*CD86*-P, mutant-type *CD86* promoter (without p-SMAD3 binding motif); SMAD3, mothers against decapentaplegic homolog 3; p-SMAD3, phosphorylated SMAD3;. HA, HA tag, CD11c-Δ; Flag, Flag-SMAD3; TGFBR1, transforming growth factor beta receptor I; IB, Immunoblot; IP, Immunoprecipitation; TGFβ1, transforming growth factor beta 1; Ctrl, control.

Liao et al. Supplementary Figure S8

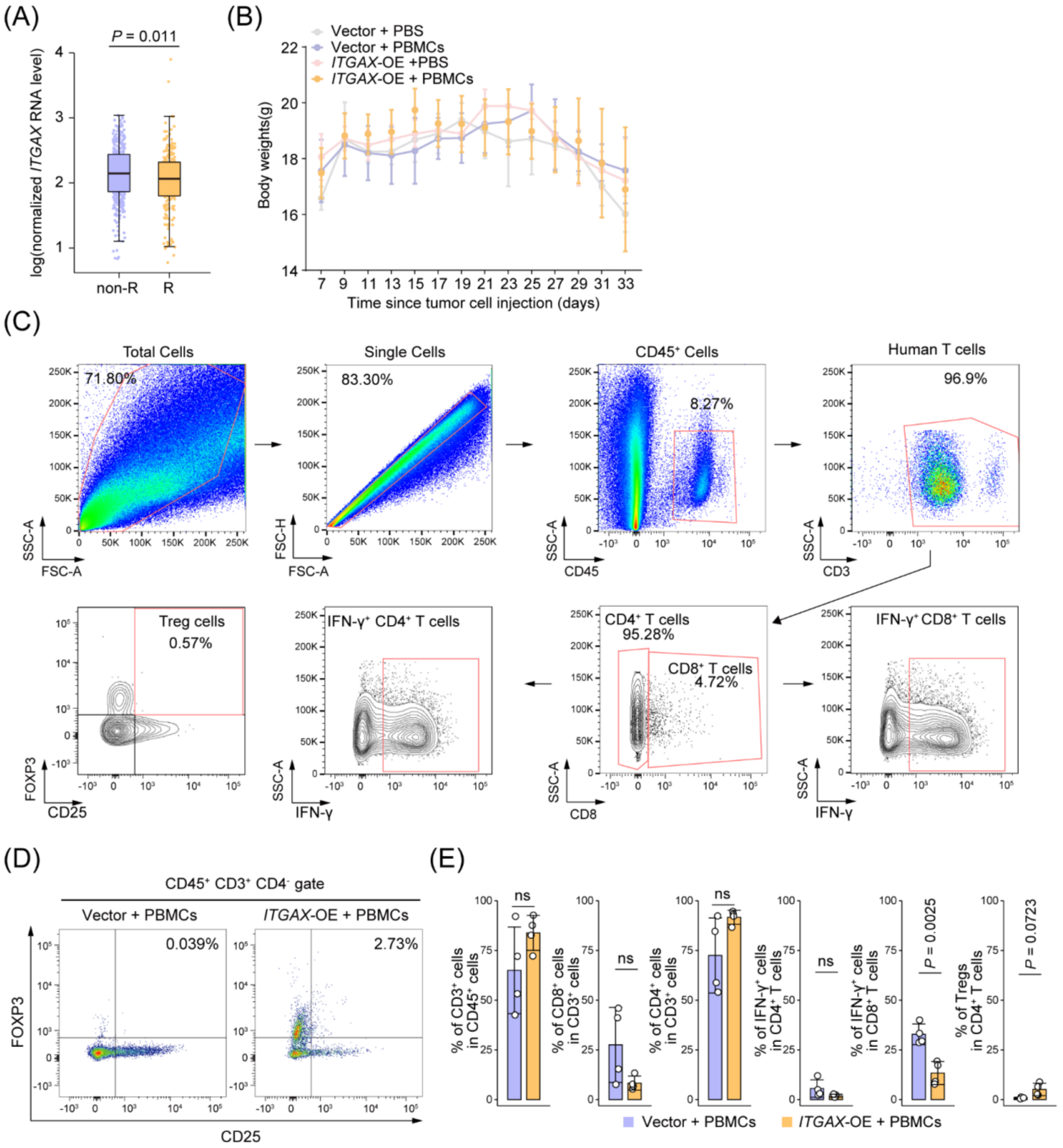

Supplementary Figure S8. CD11c promotes ESCC cell lung metastasis and immune suppressive tumor microenvironment formation. (A) Boxplots display the normalized *ITGAX* RNA levels determined by scRNA-seq in ESCC cells in patients responsive (R, n = 4) or non-responsive (non-R, n = 2) to anti-PD-L1 therapy. **(B)** Body weight curves of mice bearing lung metastasis and infused with human PBMCs. Each data represents the mean ± SD from 5 to 6 mice, related to Figure 5A. **(C)** Flow cytometry gating and analyzing strategy for IFN-γ^+CD4$^+$ T cells and CD25$^+$FOXP3$^+$ Treg cells in lung metastasis tissues of mice infused with human PBMCs. **(D)** Flow cytometry analysis of Treg cells in the CD4$^+$ T cell population isolated from mice infused with human PBMCs, related to Figure 5A. **(E)** Fractions of CD3$^+$CD45$^+$ T cells, CD3$^+$CD8$^+$ T cells, CD3$^+$CD4$^+$ T cells, IFN-γ $^+$CD4$^+$ T cells, IFN-γ$^+$CD8$^+$ T cells, and Treg CD4$^+$ cells identified by flow cytometry assays (4 mice per group), related to Figure 5A. Abbreviations: R, responsive; non-R, non-responsive; Vector, control for OE group; *ITGAX*, integrin alpha X; OE, overexpression; PBS, phosphate-buffered saline; PBMC, peripheral blood mononuclear cells; PD-L1, programmed death-ligand 1; IFN-γ, interferon gamma; FOXP3, Forkhead Box P3.

Liao et al. Supplementary Figure S9

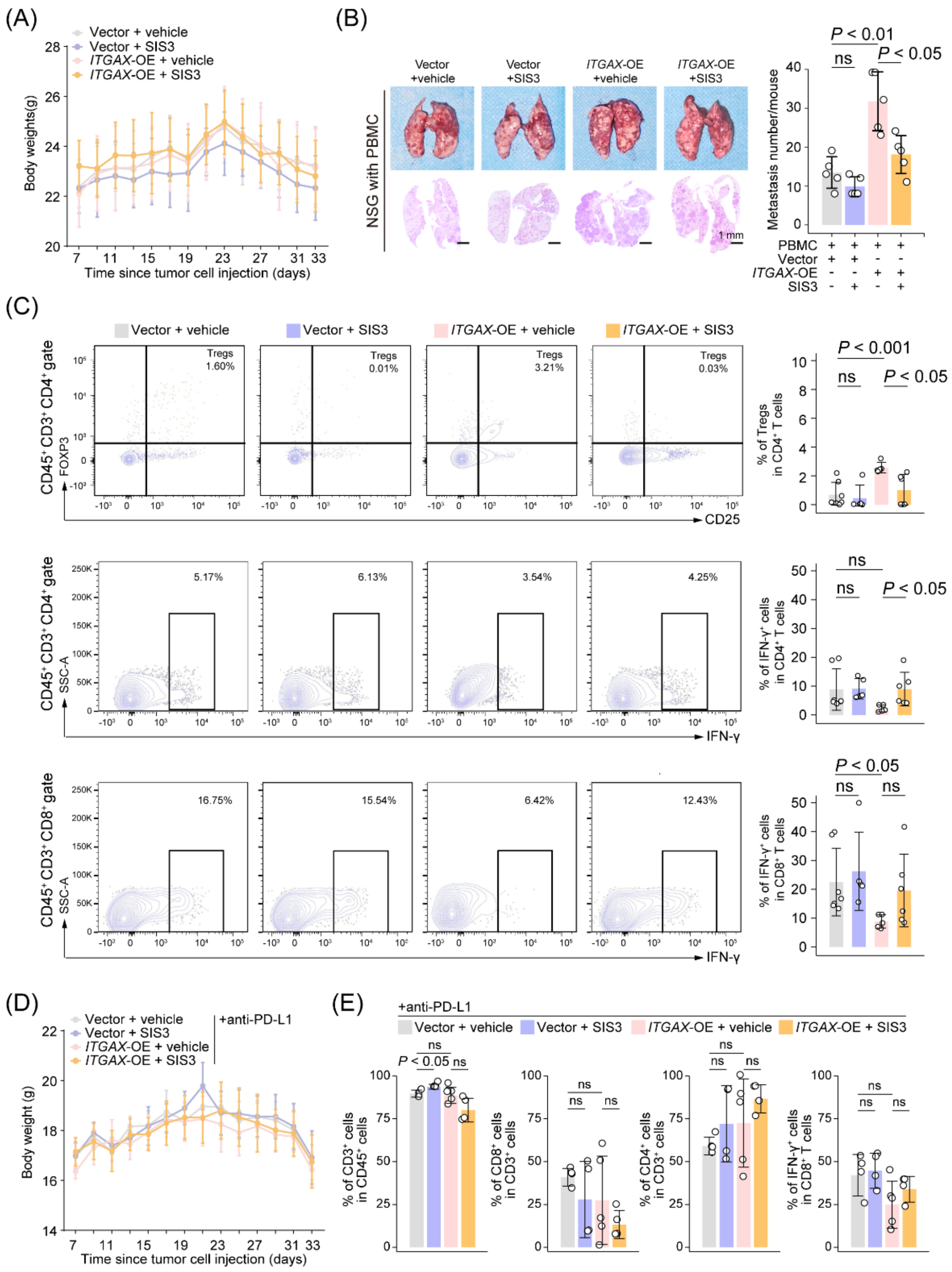

Supplementary Figure S9. Combined anti-PD-L1 and SIS3 therapy promoted a more robust anti-tumor immune response in CD11c-expressing tumors. (A) Body weight curves of humanized mice bearing lung metastases and treated with or without SIS3. Each data

represents the mean ± SD from 5 to 7 mice. **(B)** Left, representative gross and H&E staining images of lung metastases. Right, quantitative statistics of lung metastases. **(C)** Fractions of Tregs, IFN-γ^{+}CD4^{+} T cells and IFN-γ^{+}CD8^{+} T cells identified by flow cytometry assay. **(D)** Body weight curves of humanized mice bearing lung metastases and treated with anti-PD-L1 and SIS3. Each data represents mean ± SD from 5 mice, related to Figure 5B. **(E)** Fractions of CD3^{+} T cells, CD3^{+}CD8^{+} T cells, CD3^{+}CD4^{+} T cells, and IFN-γ^{+}CD8^{+} T cells identified by flow cytometry assays, related to Figure 5B–D. The data present mean ± SD from 4 to 5 mice. *P* values were from Student's *t*-test. ns, not significant. Abbreviations: Vector, control for OE group; *ITGAX*, integrin alpha X; OE, overexpression; i.v., intravenous injection; i.p., intraperitoneal injection; PBS, phosphate buffered saline; PBMC, peripheral blood mononuclear cells; PD-L1, programmed death-ligand 1; IFN-γ, interferon-gamma; FOXP3, Forkhead Box P3.

Supplementary Tables

Supplementary Table S1. Antibodies used in this study

Supplementary Table S2. Sequences of siRNAs, sgRNAs, and primers

Supplementary Table S3. Summary of mouse samples enrolled in multiplexed immunofluorescence assay

Supplementary Table S4. Top 12 gene enrichment pathways in spatial transcriptomic data

Supplementary Table S5. Gene lists used for calculating program scores

Supplementary Table S1. Antibodies used in this study

Antibody against	RRID	Applications	Dilution	Company	Catalog number
pan cytokeratin	AB_10674321	mIF	1:100	Abcam	ab86734
CD68	AB_307338	mIF	1:1,000	Abcam	ab955
Mannose Receptor	AB_1523910	mIF	1:5,000	Abcam	ab64693
CD11c	AB_2129793	mIF	1:250	Abcam	ab52632
CD8 alpha	AB_2860566	mIF	1:1,000	Abcam	ab209775
CD4	AB_2686917	mIF	1:1,000	Abcam	ab183685
CD3 epsilon	AB_443425	mIF	1:150	Abcam	ab16669
FOXP3	AB_2860568	mIF	1:100	Abcam	ab215206
CD11b	NA	mIF	1:100	eBioscience	14-0112-82
F4/80	AB_2799771	mIF	1:500	Cell Signaling Technology	70076
CD80	AB_2928982	mIF	1:1,000	Abcam	ab134120
CD86	AB_627200	mIF	1:50	Santa Cruz	sc-28347
E-Cadherin	AB_2291471	Immunoblot	1:1,000	Cell Signaling Technology	3195
N-Cadherin	AB_2687616	Immunoblot	1:1,000	Cell Signaling Technology	13116
ZO-1	AB_10898025	Immunoblot	1:1,000	Cell Signaling Technology	8193
β -Catenin	AB_11127855	Immunoblot	1:1,000	Cell Signaling Technology	8480
Vimentin	AB_10695459	Immunoblot	1:1,000	Cell Signaling Technology	5741
Slug	AB_2239535	Immunoblot	1:1,000	Cell Signaling Technology	9585
Snail	AB_2255011	Immunoblot	1:1,000	Cell Signaling Technology	3879
HLA-DR/DP/DQ/DX	AB_629967	Immunoblot	1:500	Santa Cruz	sc-53302
B7-1	AB_10987889	Immunoblot	1:500	Santa Cruz	sc-376012
B7-2	AB_627201	Immunoblot	1:500	Santa Cruz	sc-19617
MHC class I	AB_627934	Immunoblot	1:1,000	Santa Cruz	sc-32235
GAPDH	AB_2107436	Immunoblot	1:50,000	Proteintech	60004-1-Ig
SMAD3	AB_2193182	Immunoblot	1:1,000	Cell Signaling Technology	9523
Phospho-SMAD3 (Ser423/425)	AB_2193207	Immunoblot	1:1,000	Cell Signaling Technology	9520
TGFβ Receptor I/TGFBR1	NA	Immunoblot	1:500	Santa Cruz	sc-518018
Lamin B1	AB_2616597	Immunoblot	1:1,000	Abcam	ab133741
CD11c	AB_2881379	Immunoblot	1:1,000	Proteintech	60258-1-Ig
SMAD3	AB_2881879	Immunoblot	1:2,000	Proteintech	66516-1-Ig
FLAG	AB_262044	IP	1:100	Sigma-Aldrich	F1804
normal mouse IgG	AB_737182	IP	5 μg per test	Santa Cruz	sc-2025
Na,K-ATPase	AB_1310695	Immunoblot	1: 100,000	Abcam	ab76020
β-actin	AB_2687938	Immunoblot	1:10,000	Proteintech	66009-1-Ig
Rabbit IgG(H+L)-HRP	AB_3083002	Immunoblot	1:20,000	EASYBIO	BE0101
Mouse IgG(H+L)-HRP	AB_2923205	Immunoblot	1:20,000	EASYBIO	BE0102

pan Cytokeratin	AB_870750	Flow cytometry	2 μL per test	Abcam	ab52460
APC CD45	AB_10667894	Flow cytometry	5 μL per test	eBioscience	17-0459-42
FITC CD11c	AB_464940	Flow cytometry	5 μL per test	eBioscience	11-0114-82
human CD45	AB_2687376	Flow cytometry	5 μL per test	BioLegend	#368526
PerCP/Cyanine5.5 anti-human CD3	AB_893299	Flow cytometry	5 μL per test	BioLegend	#300430
APC/Cyanine7 anti-human CD8a	AB_10613636	Flow cytometry	5 μL per test	BioLegend	#300926
FITC anti-human CD4	AB_571951	Flow cytometry	5 μL per test	BioLegend	#317408
APC anti-human CD25	AB_2561976	Flow cytometry	5 μL per test	BioLegend	#356110
PE anti-human FOXP3	AB_492986	Flow cytometry	5 μL per test	BioLegend	#320108
PerCP/Cyanine5.5 anti-human IL-10	AB_315233	Flow cytometry	5 μL per test	BioLegend	#501418
PE anti-human IFN-γ	AB_315234	Flow cytometry	5 μL per test	BioLegend	#502509

Abbreviations: IP, mIF, NA

Supplementary Table S2. Sequences of siRNAs, sgRNAs and primers

**For *ITGAX* knockout**	**Sequence (5'→3')**
sg*ITGAX* #1	GGAGCTGACAGCCTTCCGTG
sg*ITGAX* #2	CAAACCCAGCGCTGTCCACA
sg*ITGAX* #3	CTGTGAGCCCATCGGCCTGC
**For *SMAD3* knockdown**	**Sequence (5'→3')**
si*SMAD3*	GAGCCTGGTCAAGAAACTCAA
**For qRT-PCR**	**Sequence (5'→3')**
GAPDH-F	GTCACCAGGGCTGCTTTTAACTC
GAPDH-R	CAGCATCGCCCCACTTGATTTTG
ITGAX-F	CTGCAAGGGTTTACATACACGG
ITGAX-R	GAATTTTGGCGGCATCCCTAC
CDH1-F	CCCTTCACAGCAGAACTAAC
CDH1-R	ACACACGCTGACCTCTAA
CDH2-F	GCTCCCTTAATTCCTCAAGTAG
CDH2-R	TCATCACCTCCACCATACA
VIM-F	CTGAACCTGAGGGAAACTAATC
VIM-R	CGTTGATAACCTGTCCATCTC
CLDN1-F	CCTCCTGGGAGTGATAGCAAT
CLDN1-R	GGCAACTAAAATAGCCAGACCT
SNAI1-F	CCCACTCAGATGTCAAGAAG
SNAI1-R	GCAGGTATGGAGAGGAAG
SNAI2-F	TCCTGGTCAAGAAGCATTTC
SNAI2-R	GTATGACAGGCATGGAGTAAC
SMAD2-F	CGTCCATCTTGCCATTCACG
SMAD2-R	CTCAAGCTCATCTAATCGTCCTG
SMAD3-F	TGGACGCAGGTTCTCCAAAC
SMAD3-R	CCGGCTCGCAGTAGGTAAC
TGFBR1-F	ACGGCGTTACAGTGTTTCTG
TGFBR1-R	GCACATACAAACGGCCTATCTC
TGFBR2-F	GTAGCTCTGATGAGTGCAATGAC
TGFBR2-R	CAGATATGGCAACTCCCAGTG
HLA-DPA1-F	ATGCGCCCTGAAGACAGAATG
HLA-DPA1-R	ACACATGGTCCGCCTTGATG
HLA-DQA1-F	TCGCTCTGACCACCGTGAT
HLA-DQA1-R	AGGGACCGTAAAACTGGTACAA
HLA-DRA-F	AGTCCCTGTGCTAGGATTTTTCA
HLA-DRA-R	ACATAAACTCGCCTGATTGGTC
HLA-A-F	ACCCTCGTCCTGCTACTCTC
HLA-A-R	CTGTCTCCTCGTCCCAATACT
HLA-B-F	CAGTTCGTGAGGTTCGACAG
HLA-B-R	CAGCCGTACATGCTCTGGA
B2M-F	GAGGCTATCCAGCGTACTCCA
B2M-R	CGGCAGGCATACTCATCTTTT

**For ChIP-qPCR**	**Sequence (5'→3')**
CD80-promoter-F	CCATCTCACCTCCAGTGTCC
CD80-promoter-R	TGTGTCTAGGAGCATGACCTG
CD86-promoter-F	TTCTTGCCTGCTTGGTGACATA
CD86-promoter-R	AAACAACTTGGCATCTTACTCAACC

Abbreviations: *ITGAX*, integrin alpha X; sgCtrl, single guide RNA control; sg*ITGAX*, single guide RNA targeting *ITGAX* gene; F, forward sequence; R, reverse sequence.

Supplementary Table S3. Summary of mouse samples enrolled in multiplexed immunofluorescence assays

Stage	Sample number	Cell number	Stroma cells	Epithelial cells
Panel 1 (epithelial and myeloid cells)				
NOR	52	107,240	37,080	70,160
INF	27	82,404	30,010	52,394
LGIN	21	65,734	24,866	40,868
HGIN	10	20,641	6,601	14,040
ESCC	13	41,782	3,357	38,425
Panel 2 (T cells)				
NOR	45	104,031	NA	NA
INF	21	48,965	NA	NA
LGIN	15	50,306	NA	NA
HGIN	6	22,521	NA	NA
ESCC	10	75,562	NA	NA

Abbreviations: NOR, normal tissues; INF, inflammatory tissues; LGIN, low-grade intraepithelial neoplasm; HGIN, high-grade intraepithelial neoplasm; ESCC, esophageal squamous-cell carcinoma.

Supplementary Table S4. Top 12 gene enrichment pathways in spatial transcriptomic data

ID	*P*	Adjusted *P*	q-value	Gene
REACTOME_INTERFERON_SIGNALING	2.664E-11	9.752E-09	5.604E-09	*EIF4G1/EIF4A2/HLA-A/HLA-E/HLA-B/IFITM3/STAT2/UBC/B2M/EIF4A1/UBB/SOCS3*
KEGG_ANTIGEN_PROCESSING_AND_PRESENTATION	2.146E-09	2.618E-07	1.505E-07	*HLA-A/HLA-E/HLA-B/TAPBP/HSP90AB1/CTSB/HSP90AA1/B2M*
REACTOME_ANTIGEN_PROCESSING_CROSS_PRESENTATION	9.506E-09	8.029E-07	4.615E-07	*HLA-A/HLA-E/HLA-B/TAPBP/UBC/B2M/UBB/PSMB3*
REACTOME_TGF_BETA_RECEPTOR_SIGNALING_IN_EMT_EPITHELIAL_TO_MESENCHYMAL_TRANSITION	3.230E-05	5.747E-04	3.303E-04	*F11R/UBC/UBB*
REACTOME_INTERFERON_GAMMA_SIGNALING	3.414E-05	5.950E-04	3.420E-04	*HLA-A/HLA-E/HLA-B/B2M/SOCS3*
KEGG_ALLOGRAFT_REJECTION	4.220E-04	4.271E-03	2.455E-03	*HLA-A/HLA-E/HLA-B*
GOMF_MHC_CLASS_I_PROTEIN_BINDING	2.357E-06	4.015E-04	3.259E-04	*HLA-E/TUBB/TAPBP/ATP5F1B*
GOMF_MHC_CLASS_II_PROTEIN_COMPLEX_BINDING	5.562E-06	7.500E-04	6.087E-04	*HSP90AB1/HSP90AA1/B2M/YWHAE*
HALLMARK_MYC_TARGETS_V1	1.427E-08	4.852E-07	4.206E-07	*HNRNPU/HNRNPA3/HSPD1/HNRNPD/HSP90AB1/HNRNPA2B1/PPIA/DDX21/LDHA/PTGES3/TUFM/YWHAE/EIF4A1/PSMB3/PGK1*
HALLMARK_UNFOLDED_PROTEIN_RESPONSE	7.709E-05	1.310E-03	1.136E-03	*SERP1/EIF4G1/EIF4A2/YWHAZ/HSP90B1/FUS/EIF4A1/KHSRP*
HALLMARK_INTERFERON_GAMMA_RESPONSE	8.385E-04	9.503E-03	8.238E-03	*HLA-A/HLA-B/TAPBP/LY6E/IFITM3/STAT2/B2M/SOCS3/LGALS3BP*
HALLMARK_MTORC1_SIGNALING	3.512E-03	2.985E-02	2.587E-02	*ENO1/PRDX1/HSPD1/SERP1/PPIA/LDHA/HSP90B1/PGK1*

Supplementary Table S5. Gene lists used for calculating program scores

For epithelial score					
A2ML1	*C1QBP*	*CRABP2*	*DDX49*	*GJB6*	*IL36G*
AAMP	*C20orf24*	*CRYAB*	*DEGS1*	*GLTP*	*IMP4*
ACAT2	*CA2*	*CSRP2*	*DHRS3*	*GLUL*	*IMPDH2*
ACOT7	*CA9*	*CSTA*	*DIMT1*	*GPNMB*	*IRF6*
ACSL1	*CALML3*	*CSTB*	*DMKN*	*GPR87*	*ISG15*
ADH7	*CALML5*	*CTDSPL2*	*DRG1*	*GPR89A*	*ITGB4*
ADIRF	*CAP1*	*EIF3K*	*DSC2*	*GPRC5A*	*JMJD7-PLA2G4B*
ADPRHL2	*CAPN2*	*EIF4EBP1*	*DSC3*	*GPS1*	*JOSD2*
ADRM1	*CCDC109B*	*EIF6*	*DSG3*	*GPS2*	*JUP*
AFG3L2	*CCT7*	*ELF3*	*DSP*	*GRB7*	*KANK1*
AHCY	*CD24*	*ELK3*	*DUOXA1*	*GRHL1*	*KIFC3*
AKR1B10	*CD46*	*EMC6*	*DUSP10*	*GRHL3*	*KLC3*
ALDH1A3	*CD55*	*EMP1*	*ECE2*	*GSTA4*	*KLF5*
ALDH2	*CD68*	*EPCAM*	*ECSIT*	*GTPBP4*	*KLK10*
ALDH3B2	*CD82*	*ERV3-1*	*EHF*	*H1F0*	*KLK11*
ALG3	*CDA*	*EXOSC4*	*EIF3I*	*HAS3*	*KLK13*
ANGPTL4	*CDC42EP4*	*FABP4*	*FGFBP1*	*HIST1H1C*	*KLK5*
ANXA1	*CDH1*	*FABP5*	*FIS1*	*HIST1H2AC*	*KLK6*
ANXA3	*CDKN1A*	*FAM213A*	*FOS*	*HIST1H4H*	*KLK7*
APOBEC3A	*CEACAM5*	*FAM57A*	*FOSL1*	*HIST2H2AA3*	*KLK8*
APOBEC3B	*CEACAM6*	*FAM83A*	*FRMD8*	*HIST2H2AA4*	*KLK9*
APRT	*CECR5*	*FBXO6*	*FTH1*	*HMOX2*	*KRT13*
AQP3	*CFB*	*FDCSP*	*FXYD5*	*HSD17B10*	*KRT14*
ATL2	*CFI*	*FDXR*	*GAA*	*HSPB8*	*KRT16*
ATP5G1	*CHCHD1*	*CTNNBL1*	*GABRP*	*HSPH1*	*KRT17*
B4GALT4	*CHMP2A*	*CTSA*	*GADD45GIP1*	*IAH1*	*KRT19*
BCL6	*CKMT1A*	*CTSC*	*GALK1*	*ID1*	*KRT23*
BHLHE40	*CLDN1*	*CYB561*	*GAST*	*IDH2*	*KRT5*
BIK	*CLDN4*	*CYB5A*	*GBA*	*IFI30*	*KRT6A*
BNIPL	*CLDN5*	*CYP26A1*	*GBP2*	*IFI6*	*KRT6B*
C10orf99	*CLDN7*	*DAPL1*	*GBP6*	*IFITM1*	*KRT6C*
C12orf10	*CLIC3*	*DAXX*	*GCNT3*	*IGFL1*	*KRT7*
C12orf75	*CLTB*	*DBI*	*GIPC1*	*IGFL2*	*KRT75*
C19orf33	*CNFN*	*DBNDD2*	*GJB2*	*IL1R2*	*KRTDAP*
C1GALT1C1	*COA4*	*DCXR*	*GJB3*	*IL1RN*	*KYNU*
C1orf116	*COQ9*	*DDX39A*	*GJB5*	*IL20RB*	*LAD1*
A2ML1	*C1QBP*	*CRABP2*	*DDX49*	*GJB6*	*IL36G*
LBH	*MYL12A*	*PI3*	*RHOV*	*SPINT1*	*TOMM40*

LCN2	*NAA20*	*PIGC*	*RPL21*	*SPNS1*	*TOMM6*
LEMD1	*NCCRP1*	*PIK3IP1*	*RPL26L1*	*SPRR1A*	*TRAP1*
LGALS3	*NDRG1*	*PIM1*	*RWDD2B*	*SPRR1B*	*TRAPPC5*
LGALS7	*NDRG2*	*PKP1*	*S100A14*	*SPRR2A*	*TRIM29*
LGALS7B	*NDRG4*	*PKP3*	*S100A16*	*SPRR2D*	*TSPAN1*
LSM10	*NDUFA4L2*	*PLK3*	*S100A2*	*SPRR2E*	*TUBA1C*
LY6D	*NDUFS7*	*PLPP1*	*S100A7*	*SPRR3*	*TUBA4A*
LYPD3	*NDUFS8*	*PLTP*	*S100A8*	*SPSB3*	*TUBB6*
MAF1	*NEAT1*	*POLDIP2*	*S100A9*	*SQRDL*	*TXN2*
MAFF	*NELL2*	*PPIF*	*S100P*	*SRD5A1*	*TXNIP*
MAL2	*NEU1*	*PPL*	*SAA1*	*SSBP1*	*TXNRD1*
MALAT1	*NFKBIL1*	*PPME1*	*SAA2*	*SULT2B1*	*TYMP*
MALL	*NME1*	*PRMT5*	*SBSN*	*TACSTD2*	*UBE2L6*
MFSD5	*NME2*	*PROS1*	*SCNN1A*	*TBCC*	*UCKL1*
MMADHC	*NMU*	*PRSS3*	*SCO2*	*TBRG4*	*UPK3BL*
MMP13	*NOL3*	*PRSS8*	*SDCBP2*	*TGFA*	*UQCRC2*
MPG	*NOP16*	*PSCA*	*SDR16C5*	*TGM1*	*UQCRFS1*
MPZL2	*NPEPPS*	*PSMC5*	*SEPHS2*	*THBD*	*VBP1*
MRPL12	*NR4A1*	*PSMD13*	*SERPINB1*	*TK1*	*VPS25*
MRPL14	*NRP1*	*PSMD6*	*SERPINB2*	*TLCD1*	*VSNL1*
MRPL16	*NSG1*	*PSMD8*	*SERPINB3*	*TMBIM1*	*WARS*
MRPL20	*NUPR1*	*PTGES*	*SERPINB4*	*TMEM109*	*WBSCR22*
MRPL21	*OAS1*	*PVRL4*	*SERPINB5*	*TMEM179B*	*WDR4*
MRPL23	*OVOL1*	*RAB11FIP1*	*SERPING1*	*TMEM40*	*WDR74*
MRPL24	*PA2G4*	*RAB25*	*SFN*	*TMEM45A*	*XPNPEP1*
MRPL54	*PCIF1*	*RAB38*	*SFXN4*	*TMEM54*	*YDJC*
MRPS12	*PDHB*	*RAB3D*	*SLC25A1*	*TMEM79*	*ZFAND6*
MRPS17	*PDZK1IP1*	*RAB9A*	*SLC39A6*	*TMEM91*	*ZFP36*
MRPS24	*PERP*	*RABGEF1*	*SLPI*	*TMPRSS11A*	*ZNF750*
MT1X	*PFKFB3*	*RAET1G*	*SMAGP*	*TMPRSS11D*	
MUC4	*PGD*	*RAP2B*	*SMIM14*	*TMPRSS11E*	
MX1	*PHB2*	*RHCG*	*SNRNP40*	*TMPRSS4*	
MXD1	*PHLDA2*	*RHOD*	*SNU13*	*TNFRSF18*	
**For partial epithelial-mesenchymal transition (pEMT) score**					
ACKR3	*COPB2*	*GJA1*	*MAGED2*	*PRSS23*	*SNAI2*
ACTN1	*CXCL14*	*HERPUD1*	*MFAP2*	*PSAP*	*STON2*
ANXA5	*DFNA5*	*HTRA1*	*MMP10*	*PSMD2*	*TAGLN*
ANXA8L1	*DHRS7*	*IGFBP3*	*MPZL1*	*PTHLH*	*TAX1BP3*
APP	*DKK3*	*IGFBP7*	*MT2A*	*PTK7*	*TGFBI*
ARPC1B	*DSG2*	*IL32*	*MYH9*	*RABAC1*	*THBS1*
BMP1	*ECM1*	*INHBA*	*NAGK*	*RTEL1-TNFRSF6B*	*TIMP3*
C1S	*EMP3*	*ITGA5*	*NMRK1*	*SEC13*	*TMED9*

CALU	*ESYT1*	*ITGA6*	*OCIAD2*	*SEMA3C*	*TNC*
CAV1	*EXT2*	*ITGB1*	*P3H2*	*SERINC1*	*TNFRSF12A*
CD63	*F3*	*ITGB6*	*P4HA2*	*SERPINE1*	*TNFRSF6B*
CD99	*FHL2*	*LAMA3*	*PDLIM7*	*SERPINE2*	*TPM1*
CDH13	*FKBP9*	*LAMB3*	*PDPN*	*SERPINH1*	*TPM4*
COL17A1	*FRMD6*	*LAMC2*	*PLAU*	*SLC31A2*	*TPST1*
COL1A1	*FSTL1*	*LGALS1*	*PLOD2*	*SLC38A5*	
COL4A2	*FSTL3*	*LTBP1*	*PLOD3*	*SLC39A14*	
COL5A2	*GALNT2*	*MAGED1*	*PRKCDBP*	*SLC7A8*	
**For mesenchymal score**					
ABI3BP	*COL8A2*	*FGF2*	*LOX*	*P3H1*	*SLIT3*
ACTA2	*COLGALT1*	*FLNA*	*LOXL1*	*PCOLCE*	*SNAI1*
ADAM12	*COMP*	*FMOD*	*LOXL2*	*PCOLCE2*	*SNTB1*
ANPEP	*COPA*	*FN1*	*LRP1*	*PDGFRB*	*SPARC*
APLP1	*CRLF1*	*FOXC2*	*LRRC15*	*PDLIM4*	*SPOCK1*
AREG	*CTGF*	*FUCA1*	*LUM*	*PFN2*	*SPP1*
BASP1	*CTHRC1*	*FZD8*	*MAGEE1*	*PLOD1*	*TFPI2*
BDNF	*CXCL12*	*GADD45A*	*MATN2*	*PMEPA1*	*TGFB1*
BGN	*CXCL6*	*GADD45B*	*MATN3*	*PMP22*	*TGFBR3*
CADM1	*CYR61*	*GAS1*	*MCM7*	*POSTN*	*TGM2*
CALD1	*DAB2*	*GEM*	*MEST*	*PPIB*	*THBS2*
CAP2	*DCN*	*GLIPR1*	*MFAP5*	*PRRX1*	*THY1*
CD44	*DKK1*	*GPC1*	*MGP*	*PRSS2*	*TIMP1*
CD59	*DPYSL3*	*GPX7*	*MMP1*	*PTX3*	*TJP1*
CDH11	*DST*	*GREM1*	*MMP14*	*PVR*	*TNFRSF11B*
CDH2	*ECM2*	*ID2*	*MMP19*	*QSOX1*	*TPM2*
CDH6	*EDIL3*	*IGFBP2*	*MMP2*	*RGS4*	*TWIST1*
COL11A1	*EFEMP2*	*IGFBP4*	*MMP3*	*RHOB*	*TWIST2*
COL12A1	*ELN*	*IL15*	*MSX1*	*SCG2*	*VCAM1*
COL16A1	*ENO2*	*IL6*	*MXRA5*	*SDC1*	*VCAN*
COL1A2	*FAP*	*ITGA2*	*MYL9*	*SDC4*	*VEGFA*
COL3A1	*FAS*	*ITGAV*	*MYLK*	*SFRP1*	*VEGFC*
COL4A1	*FBLN1*	*ITGB3*	*NID2*	*SFRP4*	*VIM*
COL5A1	*FBLN2*	*ITGB5*	*NNMT*	*SGCB*	*WIPF1*
COL5A3	*FBLN5*	*JUN*	*NOTCH2*	*SGCD*	*WNT5A*
COL6A2	*FBN1*	*LAMA1*	*NT5E*	*SGCG*	*ZEB1*
COL6A3	*FBN2*	*LAMA2*	*NTM*	*SLC6A8*	
COL7A1	*FERMT2*	*LAMC1*	*OXTR*	*SLIT2*	
**For antigen presentation (AP) score**					
B2M	*CST3*	*CXCL3*	*HLA-DPB1*	*IFI6*	*LAMC2*
BCAM	*CTSB*	*DST*	*HLA-DQA1*	*IGFBP2*	*MIA*
C1S	*CXCL10*	*GPNMB*	*HLA-DRA*	*IGFBP6*	*MT2A*

CD74	*CXCL14*	*HLA-B*	*HLA-DRB1*	*IL32*	*SAA1*
COL17A1	*CXCL2*	*HLA-DPA1*	*HLA-DRB5*	*ISG15*	*UBD*
